# Multiplatform Metabolomic Profiling of the Unilateral Ureteral Obstruction Murine Model of CKD

**DOI:** 10.3390/ijms26104933

**Published:** 2025-05-21

**Authors:** Paula Cuevas-Delgado, Verónica Miguel, Santiago Lamas, Coral Barbas, Francisco J. Rupérez

**Affiliations:** 1 Centre for Metabolomics and Bioanalysis (CEMBIO), School of Pharmacy, Universidad San Pablo-CEU, CEU Universities, Urbanización Montepríncipe, Boadilla del Monte, Madrid 28660, Spain; pau.cuevas.ce@ceindo.ceu.es (P.C.-D.); cbarbas@ceu.es (C.B.); 2 Program of Physiological and Pathological Processes, Centro de Biología Molecular “Severo Ochoa” (CBMSO, CSIC-UAM), c. Nicolás Cabrera 1, Madrid 28049, Spain; vmiguh00@gmail.com (V.M.); slamas@cbm.csic.es (S.L.)

**Keywords:** untargeted metabolomics, LC-MS, CE-MS, GC-MS, kidney tissue, renal fibrosis, UUO

## Abstract

In chronic kidney disease (CKD) research, animal models such as the unilateral ureteral obstruction (UUO) rodent model are crucial to understanding disease progression, particularly renal fibrosis. Despite its widespread use, the molecular mechanisms driving CKD remain incompletely understood. Given the interplay between metabolism and fibrosis, a comprehensive metabolomic analysis of UUO renal tissue is necessary. This study involved untargeted multiplatform analysis using liquid chromatography (LC), gas chromatography (GC), and capillary electrophoresis (CE) coupled with mass spectrometry (MS) to examine murine kidney tissue from the UUO model. The results highlight metabolic changes associated with tubulointerstitial fibrosis, which affect pathways such as the tricarboxylic acid (TCA) cycle, the urea cycle, and lipid metabolism. In particular, fibrosis impacts the lipidomic profile, with decreases in most lipid classes and increases in specific glycerophospholipids, hexosylceramides, and cholesterol esters. These findings demonstrate the value of a multiplatform approach in elucidating metabolic alterations in CKD, providing information on the underlying molecular mechanisms and paving the way for further research.

## 1. Introduction

Chronic kidney disease (CKD) is one of the leading public health problems worldwide, with a prevalence greater than 10% in the general population. In addition, this disease is labeled as a “silent killer” due to the lack of physical symptoms in its first stages, complicating its early diagnosis and management [1,2]. Elderly, women, racial minorities, and people with diabetes mellitus and hypertension have a higher risk of suffering CKD, which increases the mortality rate and the risk of cardiovascular diseases [3]. All of this, together with the absence of effective therapies to halt the development of this disease or reverse the damage caused, makes CKD a major challenge and a significant economic burden for health systems [4].

According to the KDIGO guidelines, “CKD is defined as the presence of structural or functional abnormalities of the kidney, for ≥3 months, with or without decrease glomerular filtration rate and kidney damage” [5]. Irrespective of its origin, CKD is marked by a gradual and irreversible decline in nephron count, microvascular damage, diminished renal regeneration capacity, oxidative stress, significant metabolic changes, and inflammation. These factors contribute to the development of tubulointerstitial fibrosis and glomerulosclerosis [4,6,7,8].

While the mechanisms involved in fibrogenesis are similar in most tissues and organs, there are particular characteristics associated with kidney fibrosis [8]. Some of these aspects have been discovered, including the mediators involved in the inflammatory response and those responsible for some of the profound alterations in renal metabolism [7,9,10]. However, despite intense scientific efforts directed to studying CKD, the precise global processes underlying its initiation and progression still need to be understood, given the consequent problems in the identification and development of potential new therapeutic approaches [4].

In this sense, analysis of the renal metabolome in CKD conditions holds the potential to obtain reveal invaluable insights into the intricate interplay between metabolism and kidney disease. The integration of omics technologies with cell-tracing animal models reveals that cell type-specific metabolic changes critically influence fibrogenic responses, and dissecting the heterogeneity of kidney cellular metabolic reprogramming is vital for understanding intercellular communication and advancing targeted therapies for CKD [11,12].

Metabolomics is a powerful tool for this purpose, as it allows us to develop a comprehensive overview of the metabolome present in a particular sample, such as kidney tissue [13]. In the literature, numerous investigations have aimed to characterize the CKD metabolome using different types of biological samples, mainly including biofluids such as plasma, serum or urine [14,15,16]. Although analysis of renal tissue would be essential for a greater understanding of the molecular mechanisms underlying CKD development, renal tissue is not the type of sample commonly used due to the difficulties in obtaining it, especially from humans, the complexity in processing it and the complications that come with the treatment of associated data [17].

Most metabolomic studies on CKD performed on renal tissue have been carried out with murine disease models [18]. A prevalent model for the study of this disease is unilateral ureteral obstruction (UUO) in rodents, since it generates progressive renal fibrosis [19]. This model mimics the characteristics of chronic obstructive nephropathy in humans in an accelerated way and exhibits specific features including tubular dilation, expansion of the interstitial space, reductions in proximal tubular mass, hypertrophy, hydronephrosis, infiltration of leukocytes, tubular cell death and the presence of fibroblast [19,20]. Since this is a unilateral model, it does not serve to evaluate global changes in renal function. However, non-obstructed kidneys can be used as an internal control. Further, the UUO model presents a low mortality rate. At 7 days, the UUO model does not yet present with excessive tubular atrophy or extracellular matrix deposition (ECM), allowing for the investigation of early injury mechanisms prior to the onset of advanced fibrotic remodeling. Of note, this has allowed us to study the molecular mechanisms of apoptosis and inflammation associated with this model [21]. Tubulointerstitial fibrosis progresses along with mitochondrial damage, disruption of energy metabolism, altered mitochondrial biogenesis and oxidative stress. Due to the importance of these metabolic alterations, a comprehensive evaluation of this model through metabolomics would be of great interest to elucidate the molecular mechanisms that promote disease progression [19,22].

Within metabolomic studies, untargeted analyses enable the comprehensive measurement of a sample’s metabolic profile in a given physiological condition, providing diverse information and facilitating the generation of new hypotheses [13]. This approach captures a “snapshot” of the kidney’s specific state and the repercussions of CKD on the overall body phenotype. Hence, the use of multiplatform analysis becomes essential to ensure maximal coverage of metabolites [13,23]. While untargeted metabolomics has been widely employed in CKD studies using various biological matrices from animal models or patients, the utilization of multiplatform untargeted analysis involving two or more analytical techniques remains relatively limited [23,24,25,26,27].

Given the widespread use of the UUO murine model for assessing CKD and renal damage, a thorough exploration of metabolic alterations in renal tissue with tubulointerstitial fibrosis is of significant interest in nephrology research.

The previous literature has reported metabolomic analyses of renal tissue under UUO model using nuclear magnetic resonance spectroscopy (^1^H-NMR), revealing metabolic pathway alterations in the TCA cycle, anaerobic glycolysis, amino acid, purine and pyrimidine metabolism, and methylamine metabolism, for example, as reflected by changes in the levels of some of their metabolites [19,28]. However, to further understand the metabolic changes that characterize the onset and progression of damage in UUO-associated renal fibrosis, it is necessary to be able to evaluate alterations in a broader spectrum of metabolite types, including the lipidome. To achieve this goal, it is necessary to apply a multiplatform strategy, which will allow us to profile the renal metabolome and lipidome under the UUO model by detecting numerous types of compounds, providing a holistic view of the renal metabolic map.

Our current analysis of renal tissue from a CKD murine model with renal interstitial fibrosis induced after seven days of UUO (UUO7d) employs a multiplatform untargeted metabolomics approach involving three separation techniques—liquid chromatography (LC), gas chromatography (GC), and capillary electrophoresis (CE)—all coupled with mass spectrometry (MS). In this way, we provide a comprehensive array of affected renal metabolites and lipids, assessing the impact of renal fibrosis on metabolomics and lipidomics profiles.

## 2. Results and Discussion

### 2.1. Kidney Multiplatform Untargeted Metabolomics

The multiplatform untargeted analysis applied to the kidney samples under the UUO7d model made it possible for us to detect the wide coverage of kidney metabolites and lipids, allowing us to thoroughly evaluate the differences between the metabolomic and lipidomic profiles of fibrotic and healthy renal tissue. After signal processing and data pre-treatment, the data matrices were composed of 355 features from LC-MS, 211 features from CE-MS, and 120 features from GC-MS, which were subjected to statistical analysis.

The principal component analysis (PCA-X) and partial least squares–discriminant analysis (PLS-DA) models (Figure S1) obtained for each technique assessed the analytical variation and the overall quality of the data by observing the clustering of the QC samples and the general distribution of the experimental samples. The supervised PLS-DA models highlighted the sample grouping and revealed the association of metabolites with fibrosis and control. The R2 and Q2 values obtained indicated that these models have a high goodness of fit and predictive quality.

**Figure 1:**
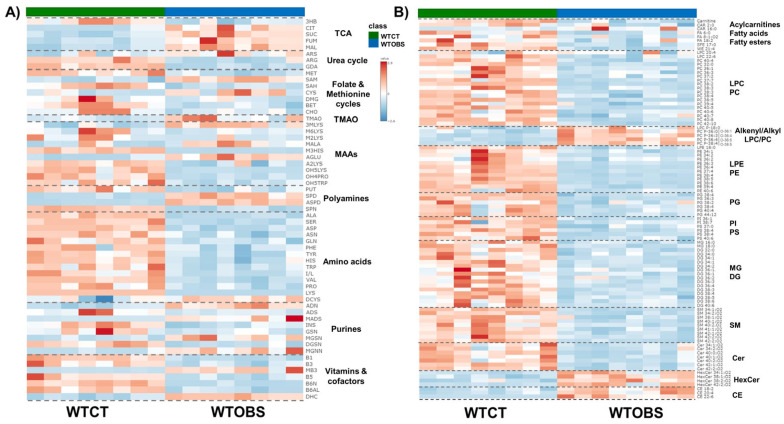
Heatmaps of significantly different metabolites and lipids detected in kidney tissue from the UUO7d model, ordered by compound ontology. (**A**) Statistically significant metabolites obtained by CE-MS and GC-MS; (**B**) statistically significant lipids obtained mainly by LC-MS. The heatmaps are based on the Euclidean distance measure and Ward clustering in Metaboanalyst 6.0 (https://www.metaboanalyst.ca; accessed on 26 August 2024); and assembled using Microsoft PowerPoint for Microsoft 365 version 2504. Abbreviations; TCA: 3HB, 3-hydroxybutyric acid; CIT, citric acid; SUC, succinic acid; FUM, fumaric acid; MAL, malic acid. Urea cycle: ARS, argininosuccinic acid; ARG, arginine; GDA, guanidinoacetate. Folate and methionine cycles: MET, methionine; SAM, S-adenosylmethionine; SAH, S-adenosylhomocysteine; CYS, cysteine; DMG, dimethylglycine; BET, glycine-betaine; CHOL, choline. TMAO: TMAO, trimethylamine-oxide. MAAs, modified amino acids: 3MLYS, N,N,N-trimethyl-lysine; M6LYS, N6-methyl-lysine; M2LYS, N2-methyl-lysine; MALA, methyl-alanine; M3HIS, 3-methyl-histidine; AGLU, acetyl-glutamic acid; A2LYS, N2-acetyl-lysine; OH4PRO,4-hydroxy-proline; OH5LYS, 5-hydroxylysine. Polyamines: PUT, putrescine; SPD, spermidine; ASPD, acetyl-spermidine; SPN, spermine. Amino acids: ALA, alanine; SER, serine; ASP, aspartic acid; ASN, asparagine; GLN, glutamine; PHE, phenylalanine; TYR, tyrosine; HIS, histidine; TRP, tryptophan; I/L, isoleucine/leucine; VAL, valine; PRO, proline; LYS, lysine; DCYS, cystine. Purines: ADN, adenine; ADS, adenosine; MADS, methyl-adenosine; INS, inosine; GSN, guanosine; MGSN, methyl-guanosine; DGSN, deoxy-guanosine; MGNN, methyl-guanine. Vitamins and cofactors: B1, thiamine; B3, niacinamide: MB3, methyl-niacinamide; B5, pantothenate; B6N, pyridoxamine; B6AL, pyridoxal; DHC, dehydroascorbic acid.

The 155 annotated compounds that are statistically significant in this study are depicted in heatmap models in Figure 1. These heatmaps facilitate the visualization of the direction of changes in the 61 metabolites (Figure 1A) and 94 lipids (Figure 1B) associated with UUO-induced fibrosis. Simultaneously, these heatmaps classify significant compounds based on biochemical ontologies and lipid categories. Detailed information regarding annotation, analytical technique, annotation confidence level, variation value and statistical significance is further elaborated in Supplementary Tables S1–S4.

There are different metabolic patterns associated with the presence of fibrosis (Figure 1). Most of the significant metabolites that present changes associated with renal fibrosis are amino acids and amino acid-related compounds, as well as metabolites that belong to essential biochemical pathways (Figure 1A). However, significantly altered lipids are not just restricted to one single lipid family but changes are also seen in fatty acyls (fatty acids, their conjugates, and fatty esters, FA), acylcarnitines, glycerophosphocholines (PC), glycerophosphoethanolamines (PE), glycerophosphoglycerols (PG), glycerophospho-serines (PS), glycerophosphoinositols (PI), monoradylglycerols (MG) diradylglycerols (DG), ceramides (Cer), glycosphingolipids (HexCer), phosphosphingolipids (SM) and sterols (CE) (Figure 1B).

Overall, nearly all metabolites and lipids are downregulated in the WTOBS group compared to the WTCT group. However, there is an exception for some compounds such as citric acid, succinic acid, fumaric acid, malic acid, argininosuccinic acid, spermidine, N1-acetylspermidine, S-adenosyl-methionine, cysteine, cystine, N,N,N-trimethyl-lysine, N-methyl-alanine, N-acetyl-glutamic acid, adenine, N-methyl-adenosine, methyl-guanine, methyl-guanosine, N1-methyl-nicotinamide, TMAO and lipids such as ether phosphocholines (PC (O-|P-)), HexCer and CE, which are highly upregulated in the fibrosis group.

In the following sections, the results derived from this study will be presented and discussed concerning the main biochemical pathways affected.

### 2.2. Renal Fibrosis Produces Dysfunction in the TCA with the Accumulation of Its Intermediates

A general increase is observed in all detected intermediates of the TCA cycle, such as succinic acid, fumaric acid and malic acid, in the presence of fibrosis, compared to the control group. Previous studies have demonstrated the crucial role of the TCA cycle in the maintenance of metabolic homeostasis and how its dysfunction or disruption, with the consequent accumulation of some of its intermediates, was linked to the activation of several stress response mechanisms [29].

**Figure 2:**
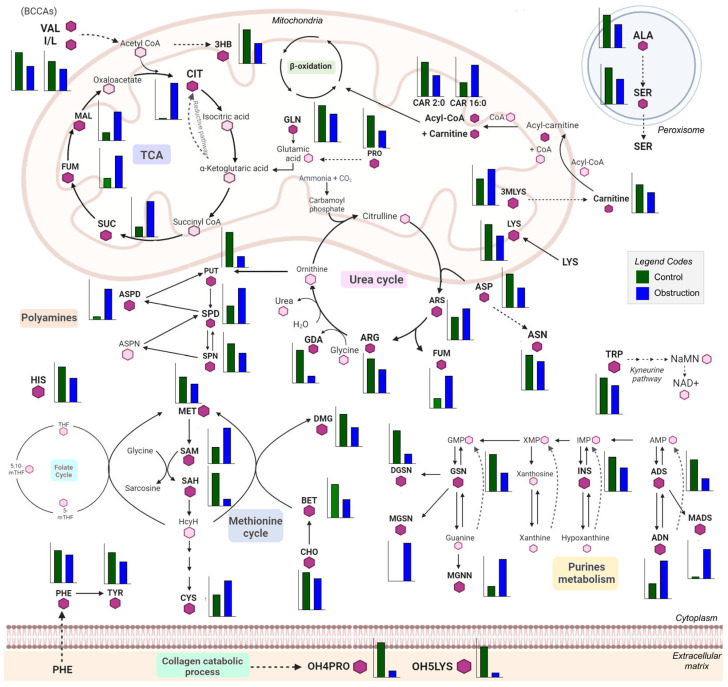
Scheme of renal metabolism depicting changes in the significantly different metabolites between fibrosis and control groups. The annotated metabolites are represented in bold letters and with a hexagon in dark purple, and they have a bar graph next to them showing the abundance of the corresponding metabolites in both experimental groups, the control group (green) and the obstruction (blue) group. Each graph presents a different scale, depending on the abundance of a given metabolite. Those metabolites present in each pathway but not annotated in the study are represented by a light-purple hexagon. The main pathways represented in this scheme are the TCA cycle, the urea cycle, polyamine metabolism, the methionine cycle, purine metabolism, amino acid metabolism and the collagen catabolic process. Bar plots were generated using GraphPad Prism 9.5.1 (GraphPad Software, San Diego, CA, USA) and the schematic was created using Microsoft PowerPoint for Microsoft 365, version 2405. Abbreviations: TCA: 3HB, 3-hydroxybutyric acid; CIT, citric acid; SUC, succinic acid; FUM, fumaric acid; MAL, malic acid. Urea cycle: ARS, argininosuccinic acid; ARG, arginine; GDA, guanidinoacetate. Folate and methionine cycles: MET, methionine; SAM, S-adenosylmethionine; SAH, S-adenosylhomocysteine; CYS, cysteine; DMG, dimethylglycine; BET, glycine-betaine; CHOL, choline. TMAO: TMAO, trimethylamine-oxide. MAAs, modified amino acids: 3MLYS, N,N,N-trimethyl-lysine; OH4PRO,4-hydroxy-proline; OH5LYS, 5-hydroxylysine. Polyamines: PUT, putrescine; SPD, spermidine; ASPD, acetyl-spermidine; SPN, spermine. Amino acids: ALA, alanine; SER, serine; ASP, aspartic acid; ASN, asparagine; GLN, glutamine; PHE, phenylalanine; TYR, tyrosine; HIS, histidine; TRP, tryptophan; I/L, isoleucine/leucine; VAL, valine; PRO, proline; LYS, lysine; DCYS, cystine. Purines: ADN, adenine; ADS, adenosine; MADS, methyl-adenosine; INS, inosine; GSN, guanosine; MGSN, methyl-guanosine; DGSN, deoxy-guanosine; MGNN, methyl-guanine.

The most remarkable increase in this comparison is shown in citric acid levels, with a 25-fold increase in the fibrotic group compared to the control (Table S1 and Figure 2). In the majority of metabolomic studies performed in animal models of renal fibrosis, notable alterations have been observed on the citric acid levels. However, the reported changes vary depending on the type of sample analyzed [27]. Thus, decreased urine citric acid levels have been associated with CKD progression [30], whereas in plasma, these levels either show an increase or remain unchanged [31]. Citric acid is the most abundant pH-sensitive metabolite present in the urine, and its reabsorption by the proximal tubule determines its urinary excretion. When the reabsorbed citric acid is converted into glucose, or CO_2_ and H_2_O, protons are consumed and HCO_3_^-^ is generated. Thus, citrate reabsorption means a base gain, but its excretion represents a base loss [30]. In a situation of renal damage or fibrosis, an acid–base disbalance occurs. Therefore, the accumulation of citric acid in the tissue points to an attempt to enhance its metabolism and balance the acid–base status to avoid an acidosis situation in the whole organism [32]. This striking increase in citrate in renal tissue is also observed in the model of renal fibrosis produced by folic acid overdose, suggesting that alterations in the TCA cycle are related to interstitial fibrosis regardless of its origin [27]. Another pathway that could be involved in the drastic increase in citrate is the reductive carboxylation produced in the TCA cycle, whereby α-ketoglutarate is produced from glutamine and converted into citrate [33]. This reductive pathway is a major cellular carbon source for fatty acid synthesis during hypoxia or mitochondrial impairment, resulting in an alteration in the α-ketoglutarate/citrate ratio [33,34]. Additionally, the significant elevation in citrate levels could be indicative of a renal homeostatic response aimed at reinstating acetyl-CoA carboxylase (ACC) activity and fatty acid synthesis, as previously postulated [27]. This is substantiated by the observed increase in the levels of phosphorylated AMP-activated protein kinase (AMPK) and ACC enzymes in prior studies on this model [35]. The role of ACC as a critical component in this pathway underscores the alterations that transpire in renal fibrosis. Therefore, the pronounced surge in citrate levels might be a compensatory mechanism to counterbalance these changes.

In addition, a significant decrease is observed in the levels of the ketone body 3-hydroxybutyric acid (3-HB) in the WTOBS group. This metabolite is produced as an alternative product of fatty acid oxidation. It is an important energy source for kidneys under energy stress and provides acetoacetyl-CoA and acetyl-CoA for synthesizing different complex lipids and starting the TCA cycle [36]. Previous studies have shown that 3-HB is beneficial in the treatment of several kidney diseases, as it reduces oxidative stress and inflammation while halting the apoptosis of kidney cells [36]. Moreover, in the UUO model, it has been shown that a ketogenic diet alleviates renal fibrosis, producing an increase in 3-HB levels, which is related to the enhancement of fatty acid oxidation (FAO), causing reductions in macrophage infiltration and allowing for the regulation of its proliferation [37,38].

### 2.3. Arginine Synthesis Is Decreased in Renal Fibrosis Associated with the Downregulation of the Urea Cycle

**Figure 3:**
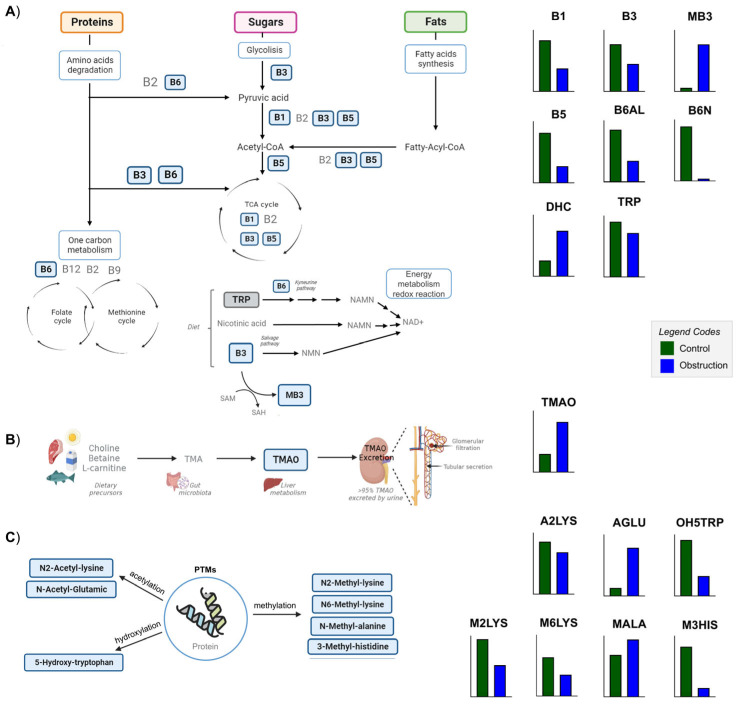
Scheme of the significantly different metabolites of B vitamin family metabolism, TMAO metabolism and modified amino acid (MAA) metabolism. The metabolites annotated in this study are represented in bold and within a blue box. Their respective abundance in the control and obstruction groups is represented in bar graphs beside the figures in green and blue, respectively. (**A**) Diagram of the role of B vitamins as cofactors in the metabolism of proteins, sugars and fats, carrying out essential functions in the degradation of amino acids, glycolysis, fatty acid synthesis, one-carbon metabolism and energy metabolism, and being involved in the synthesis of NAD+ and redox reactions. (**B**) Schematic representation of the TMAO synthesis and excretion. (**C**) Grouping of MAAs according to the PTMs of amino acids in proteins. Bar plots were generated using GraphPad Prism 9.5.1 (GraphPad Software, San Diego, CA, USA), and the figure was created and assembled using Microsoft PowerPoint for Microsoft 365, version 2405. Abbreviations: TMAO: TMAO, trimethylamine-oxide. MAAs, modified amino acids: M2LYS, N2-methyl-lysine; M6LYS, N6-methyl-lysine; MALA, methyl-alanine; M3HIS, 3-methyl-histidine; A2LYS, N2-acetyl-lysine; AGLU, acetyl-glutamic acid; OH5TRP, 5-hydroxy-tryptophan. Vitamins and cofactors: B1, thiamine; B3, niacinamide: MB3, methyl-niacinamide; B5, pantothenate; B6N, pyridoxamine; B6AL, pyridoxal; DHC, dehydroascorbic acid.

The urea cycle, also known as the ornithine cycle, involves a series of biochemical reactions that occur primarily in the liver, and which converts highly toxic ammonia into urea for its excretion, mainly by the kidneys [39]. In this pathway, we can observe arginine levels decreased in the fibrosis group compared to the control one. On the other hand, levels of argininosuccinic acid, which is produced from citrulline and is a precursor of arginine, were increased in the WTOBS group compared to the control. In addition, levels of guanidinoacetate, the synthesis of which in the kidney is dependent on the endogenous synthesis of arginine, were remarkably decreased in the fibrosis group compared to the control group (Table S1 and Figure 3).

Previous work has demonstrated that there exists a disruption in arginine metabolism and nitric oxide (NO) synthesis in CKD-associated tubulointerstitial fibrosis and other kidney diseases [27,40,41]. Specifically, in the UUO model, it has been found that nitric oxide synthase (NOS) activity is reduced, despite increasing its expression, so that NO levels in the tissue in this model of damage are lower [42]. Arginine levels in both plasma and tissue in 24-h UUO are not significantly reduced, but a trend is observed, and in a model of bilateral UO (BUO), decreases in the levels of this metabolite have been observed. These changes have a direct effect on blood flow, so they have direct consequences at the cardiovascular level, both at a systemic and renal level, and are involved in the progression of renal damage [42].

Additionally, arginine availability has been shown to regulate the mechanistic target of the rapamycin (mTOR) pathway, which plays a critical role in cell growth and fibrogenesis. In this context, the decreased arginine levels observed in fibrotic renal tissue might act as a limiting factor that counteracts mTOR activation, potentially representing a compensatory mechanism to modulate fibrosis progression [43,44].

### 2.4. Fibrosis Produces Alterations in One-Carbon Metabolism with Changes in the Levels of S-Adenosyl-Methionine and Glycine-Betaine

Renal fibrosis generates alterations in the levels of metabolites belonging to the methionine cycle, which is essential for maintaining methylation homeostasis, through the regulation of the production of methionine and S-adenosylmethionine (SAM) through the consumption of homocysteine and S-adenosylhomocysteine (SAH), the kidney being the main site of elimination of the SAH [45].

Levels of an important osmoregulatory compound, glycine-betaine, and of dimethylglycine (DMG) decreased in WTOBS compared to the control group. Also, levels of choline, a precursor of glycine-betaine, slightly decreased in the fibrosis group. Methionine and SAH were significantly decreased in the fibrosis group compared to the control. Simultaneously, SAM was highly increased in the fibrosis group, together with a slight increase in cysteine levels (Table S1 and Figure 2).

Systemic and local disruptions of methionine-dependent pathways have been observed in a murine model of folic acid nephropathy [27] and in patients with CKD [46]. Alterations in the levels of SAM, homocysteine, and cysteine in the plasma and urine of patients have been previously observed [46]. Given that kidneys play an essential role in maintaining SAH and homocysteine levels, the disruption of the filtration and reabsorption functions of kidneys, along with the dysregulation of the methionine cycle, should impact the progression of the disease [47]. In addition, glycine-betaine, a crucial metabolite in this pathway, acts as a methyl donor and osmoprotectant, also displaying an anti-inflammatory role, in the development of kidney disease [47].

Another important point to consider within these alterations is that one-carbon metabolism is one of the pathways involved in nicotinamide adenine dinucleotide (NAD^+^) biosynthesis and regulation [46]. The enzyme nicotinamide N-methyltransferase (NNMT) regulates both NAD^+^ and methionine methylation. Therefore, the accumulation of SAM indicates alterations in the expression and/or function of NNMT, which will be reflected in alterations in the synthesis, degradation, and availability of NAD^+^ and intermediates [48].

### 2.5. Renal Amino Acid Metabolism Is Compromised by Fibrosis

Overall, we observed that fibrosis induced by the UUO7d model produces a decrease in amino acid levels (Table S2 and Figure 2). The only two amino acids that show a different trend with a slight increase in their levels in the WTOBS group are cysteine and cystine.

This general decrease has been previously observed in kidney tissue with interstitial fibrosis induced by folic acid [27]. Biologically, an overall reduction in amino acid levels implies a decrease in tissue protein proteolysis and/or an increase in amino acid utilization for protein synthesis or further metabolism. In renal fibrosis, there is a well-known increase in ECM caused by an imbalance between matrix protein production and degradation. Renal tissue harbors a proteolytic network comprising various proteases, including metalloproteinases, and their regulators. This protein degradation system is impaired in the initiation and progression of renal damage and interstitial fibrosis, leading to a decline in free amino acids [49]. In addition, the activation of the mTOR signaling pathway—commonly linked to increased anabolic processes such as protein synthesis—together with alterations in AMPK signaling may contribute to enhanced amino acid consumption in fibrotic kidney tissue [50,51]. This local amino acid imbalance becomes further relevant when considering the kidneys’ pivotal role in whole-body amino acid metabolism and homeostasis. Given that the kidney is the major organ for the disposal of glutamine, proline, citrulline, cysteine–glycine, and SAH from the arterial blood, and for the net release of some amino acids such as serine, cysteine, tyrosine and arginine, renal fibrosis can induce deregulation and alterations in their levels, with a global impact in the whole system’s metabolism [52].

### 2.6. Methylated, Acetylated, and Hydroxylated Amino Acids Are Profoundly Altered in the UUO Model

Post-translational modifications (PTMs) of amino acids present in proteins and peptides are also involved in the pathogenesis of several diseases, including CKD and renal fibrosis [53]. Cleavage of post-transcriptionally modified proteins releases modified amino acids (MAAs) [54]. In this work, we detected different changes in the levels of some MAAs in kidney tissue from the UUO7d model (Table S2, Figure 2 and Figure 3C).

An important type of PTM is methylation, including histone and non-histone methylation. We detected variations in two monomethylated lysines, N2-methyl-lysine and N6-methyl-lysine (Figure 3C) and N,N,N-trimethyl-lysine (Figure 2). These methylations affect protein activity, protein–protein interactions and interactions with other PTMs. When the methylated AA residues are part of the histones, methylation is associated with gene repression or activation, whereas when non-histone proteins are methylated, various cellular mechanisms are affected [55]. In this fibrosis model, we can observe a decrease in monomethylated lysines in the WTOBS group, which is in line with previous studies in UUO where distinct patterns of histone modifications in the kidney were observed, including changes in the monomethylation and dimethylation of lysine residues in histones [56]. By contrast, in the case of trimethyl-lysine, we can see an increase in levels of it in the fibrosis group. This MAA is a precursor of carnitine in the kidney, and elevated concentrations have previously been seen in the plasma of patients with renal failure [57]. Such an increase in renal tissue could indicate reduced carnitine availability, which, together with the mitochondrial damage associated with kidney damage, would contribute to a renal energy deficit. A slight increase in methyl-alanine, along with a marked decrease in 3-methylhistidine levels, is observed in the WTOBS group compared to the control one (Figure 3C). In the case of 3-methylhistidine, alterations in its plasma and urinary concentrations have previously been observed in patients with renal damage, this amino acid being a biomarker of myofibrillar protein degradation [58].

Acetylation is another PTM that impacts protein function. Dysfunction of the enzymes involved in these reactions may be reflected in the concentration of acetylated AAs [59]. Recently, aberrant lysine acetylation has been associated with CKD, metabolic syndrome and other diseases [60]. In our data, the levels of N2-acetyl-lysine are decreased in the fibrosis compared to control condition, whereas the levels of N-acetyl-glutamic are highly increased (Figure 3C). Alterations in the expression of N-acetyltransferases in the kidney have recently been associated with different concentrations of N-acetyl amino acids in the plasma, and, in turn, have been correlated with renal failure [61]. N-acetyl-glutamic acid is the allosteric activator of the first enzyme in the urea cycle (carbamyl phosphate synthetase I (CPSI)). Therefore, an accumulation of N-acetyl-glutamic acid in the kidney tissue would lead to abnormal urea cycle function, generating hyperammonemia [62].

5-hydroxylysine and 4-hydroxyproline are hydroxylated derivatives of lysine and proline, which are uniquely present in collagen-like peptides. Their free forms arise uniquely through the proteolytic degradation of these peptides [63]. Collagen is one of the main components of the ECM, and it plays an important role in the pathogenesis of chronic fibrosis diseases. During the progression of these diseases, there are changes in collagen composition, modification, and crosslinking. Due to the turnover of extracellular collagen being very slow, these modifications accumulate more in fibrosis states [64]. In our data from the UUO7d model, the levels of 5-hydroxylysine and 4-hydroxyproline are significantly decreased in the WTOBS group (Figure 2). The levels of these MAAs are likely related to the reduced turnover and degradation of collagen within the ECM in fibrosis conditions [27]. Another hydroxylated amino acid detected in our dataset is 5-hydroxy-tryptophan, the levels of which are significantly decreased in the WTOBS group compared to the control one (Figure 3C). This MAA is the precursor of 5-hydroxytryptamine (5-HT, serotonin), produced in the renal proximal convoluted tubules through a decarboxylation reaction. Alterations in the production of 5-HT could affect renal function and glomerular structure since this compound can induce renovasoconstriction, antinatriuresis, antidiuresis and fibrosis in the kidney. Furthermore, it has been observed that proximal tubular alterations aggravate imbalances in renal 5-HT production [65].

### 2.7. Renal Fibrosis Alters Polyamine Metabolism in Kidney Tissue

Both putrescine and spermine followed a decreasing trend in the fibrosis group, although in different proportions (Table S2). By contrast, spermidine levels increased in the WTOBS group. However, the most notable change in this pathway was observed in N1-acetylspermidine, the levels of which increased nine-fold in the WTOBS group compared to the WTCT one (Table S2 and Figure 2). These results suggest that polyamine synthesis is favored, while its catabolism is blocked.

Previous studies employing various renal damage models, such as UUO, ischemia–reperfusion, and adenine-induced injuries, have reported changes in the levels of putrescine, spermine, and spermidine that align with our findings [66,67]. This suggests that kidneys respond to diverse injury types, with alterations in this biological pathway contributing to the progression of kidney damage [66,67]. Numerous studies have explored the impact of spermine or spermidine supplementation on renal damage. These investigations have demonstrated that spermine exhibits a protective role in mitigating renal ischemia/reperfusion injury (IRI) by promoting autophagy, inhibiting inflammatory reactions, and reducing oxidative stress and the endoplasmic reticulum stress response (ERSR) [68]. Additionally, polyamines undergo catabolism via the spermidine/spermine N1-acetyltransferase/N1-acetylpolyamine oxidase cascade (SSAT/PAOX) and the direct oxidation of spermine by spermine-oxidase (SMOX). The oxidation processes of these catabolic intermediates generate harmful substances such as H_2_O_2_ and aminoaldehydes, which disrupt the integrity of lysosomal and mitochondrial membranes, thereby inducing ERSR [69]. Numerous studies involving cell cultures and models of kidney damage have demonstrated an upregulation in SSAT gene expression, which is associated with fibrosis progression [70]. Furthermore, in the model of tubulointerstitial fibrosis produced by folic acid nephropathy, a large increase in the levels of N1-acetylspermidine in renal tissue has also been observed [27]. Therefore, considering the collective findings from these studies, it can be postulated that this alteration consistently manifests in the development of renal fibrosis, irrespective of the damage’s origin.

### 2.8. The Presence of Methylated Purine Derivatives Characterizes Renal Fibrosis

The purine-derived metabolites with remarkable changes in our dataset are methylated derivatives, such as methyl-adenosine, methyl-guanine and methyl-guanosine, which are highly increased in fibrosis. Notably, methyl-guanine is only present in this group (Table S3 and Figure 2). We also found increased adenine levels in the WTOBS group, while the other metabolites detected in this pathway, such as adenosine, inosine, guanosine and deoxyguanosine, were decreased.

Purine catabolism seems to be intensified in fibrosis conditions, being a pathway that is related to redox and oxidative stress (ROS) [27,71]. In the UUO model, reduced purine metabolism has previously been seen, with decreased inosine and adenosine levels [28], as well as alterations in adenine receptor expression, all these changes being directly related to the development of fibrosis and damage and being able to be modulated pharmacologically with adenosine antagonists and agonists [72]. In addition, several studies show that alterations in the levels of methylated purine metabolites and in the expression or activity of the enzymes involved in their generation or degradation are associated with various pathologies, including kidney cancer, polycystic kidney disease, acute kidney injury and obstructive renal nephropathy [73]. These methylated purine bases produce epigenetic modifications in DNA and RNA sequences, playing critical roles in the evolution of renal diseases [74].

### 2.9. Decreased Levels of B Family Vitamins Due to Renal FIBROSIS Could Be Associated with NAD^+^ Shortage

The levels of the different B vitamins are decreased in the fibrosis group compared to the control one. This applies to thiamine (vitamin B1), nicotinamide or niacinamide (vitamin B3), pantothenate (vitamin B5), and two isoforms of vitamin B6, pyridoxal and pyridoxamine (Table S3 and Figure 3A). On the other hand, levels of the methylated derivative of nicotinamide, n1-methylnicotinamide, are remarkably increased in the WTOBS compared to WTCT group. Moreover, the oxidized form of vitamin C, dehydroascorbic acid (DHC), also present increased levels in the fibrosis group compared to control one (Table S3 and Figure 3A).

Vitamins are essential natural organic compounds that play crucial roles in normal physiological functions, such as cell survival, proliferation and differentiation. In addition, they can modulate signal transduction and epigenetics [75]. Vitamins from the B family are mainly involved in the energetic metabolism pathway, participating as cofactors in glycolysis, fatty acid synthesis and degradation, amino acid catabolism, the TCA cycle and oxidative phosphorylation [76]. For this reason, any deficiency in these vitamins could lead to alterations in cell energy production [75]. Interestingly, signaling pathways activated during fibrosis, such as mTOR and NRF2, may contribute to the decreased levels of B vitamins observed in fibrotic kidneys. mTOR has been shown to upregulate the expression of enzymes and transporters involved in B-vitamin activation and utilization, while NRF2 promotes the expression of antioxidant enzymes that rely on B-vitamin cofactors. Therefore, their sustained activation in renal fibrosis could increase the cellular demand and turnover of these vitamins, leading to their depletion [77,78].

It has also been shown that patients with CKD and other kidney diseases present alterations in their plasma and the urinary levels of vitamins. Kidney diseases result in excessive losses of water-soluble vitamins via the urine because these vitamins are freely filtered by the glomerulus and can be lost in cases of tubular dysfunction [79].

Vitamin B3 is a family of compounds including nicotinic acid, niacinamide and nicotinamide riboside. They are precursors of NAD^+^, which serves as a cofactor or substrate in a wide range of metabolic and redox reactions. Due to the importance of NAD+ in metabolism, its synthesis pathways and precursors are highly regulated. Consequently, their alterations have a huge impact on the development of many diseases [75]. For instance, dysregulation of NAD^+^ metabolism contributes to the initiation and progression of CKD [48]. In acute kidney injury (AKI) patients, urine levels of tryptophan and kynurenine are decreased, suggesting reduced activity of the kynurenine pathway of NAD^+^ synthesis from tryptophan [80]. In addition, the methylation of niacinamide, through the enzyme NNMT, regulates NAD^+^ and methionine metabolism. This enzyme is expressed abundantly in the kidney, but its role in CKD and renal fibrosis remains unclear. In a study on the UUO model, renal NNMT expression and methyl-niacinamide levels were increased, while NAD^+^ and NADH precursors were decreased. Also, NNMT deficiency was demonstrated to ameliorate renal fibrosis through an increase in the DNA methylation of the connective tissue growth factor. It also improved renal inflammation by increasing renal NAD^+^ and Sirt1 levels, while decreasing NF-κβ acetylation [48]. Therefore, this B3 methylation is emerging as a key intersection between cellular metabolism and epigenetic regulation, and growing evidence supports its central role in several pathologies, including CKD [76].

Regarding the other water-soluble vitamin detected in our study, DHC, previous studies have demonstrated that renal dysfunction in CKD patients is associated with a decrease in plasma vitamin C levels [81]. Vitamin C or ascorbate acts as an electron donor and scavenger of free radicals, being a potent antioxidant. DHC is generated from these reactions due to the pro-oxidant environment generated in renal fibrosis [82]. Moreover, vitamin C regulates collagen synthesis by acting as a cofactor for enzymes involved in its generation. Elevated levels of vitamin in renal tissue are likely to contribute to the increased collagen production for ECM in renal fibrosis [75].

### 2.10. Increased Renal TMAO Levels Indicate CKD Progression

The oxidation product of trimethylamine (TMA), TMAO, was present in increased levels in the fibrosis group compared to the control one (Table S1 and Figure 3B). TMAO is produced in the liver by the oxidation of trimethylamine (TMA), a product of the gut microbiota generated from choline, betaine and L-carnitine. TMAO is mainly excreted in the urine and has previously been associated with cardiovascular diseases, kidney disease, type 2 diabetes and tumors. In the last few years, the role of TMAO in CKD progression has been gradually understood [83]. Impaired renal TMAO clearance may lead to elevated renal and circulating TMAO concentrations, which can contribute to inflammation and renal oxidative stress [83,84]. In addition, TMAO has been found to promote renal fibroblast activation and proliferation by activating the PERK/Akt/mTOR pathway, NLRP3, and caspase-1 signaling [85].

### 2.11. Renal Fibrosis Greatly Impacts the Kidney’s Lipidomic Profile

**Figure 4:**
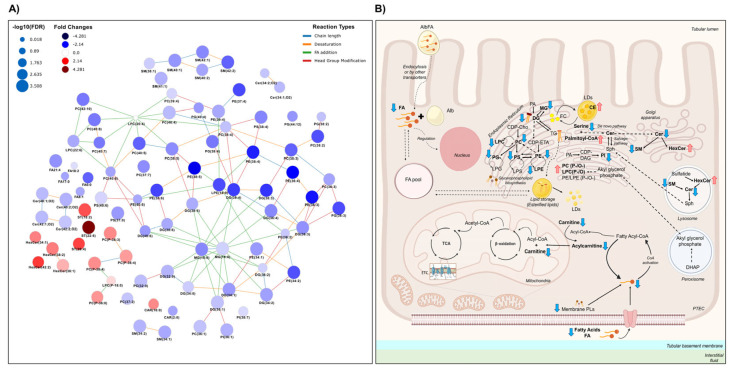
Scheme of the significantly different lipids and lipid metabolism in the UUO7d model. (**A**) Interactive lipid network in a comparison of WTOBS vs. WTCT showing the different alterations in lipid classes. This network was obtained using the LINEX web tool (https://exbio.wzw.tum.de/linex/; accessed on 26 August 2024) [86]. The node size is scaled using the negative log10 of *p*-values corrected by FDR. Colors represent fold changes: blue indicates lower levels of lipids in the obstruction group, while red indicates higher lipid levels in the obstruction group, compared to the control group in both cases. (**B**) Schematic representation of lipid metabolism in kidney proximal tubular epithelial cells (PTECs). Lipid classes detected in this study are shown in bold, and the significant changes observed are represented by blue downward or red upward arrows reflecting the decrease or increase in the obstruction group versus the control, respectively. The network was generated using LINEX, and the final figure was assembled using Microsoft PowerPoint for Microsoft 365, version 2405, with vector graphics sourced from a free, royalty-free PNG image repository.

The presence of fibrosis produces changes that are reflected in the different lipid families in the UUO7d model (Table S4 and Figure 4). We can observe that levels of most of the lipids from the different classes were decreased in the WTOBS group. However, an increase was observed in sterols, hexosylceramides and phospholipids with ether substituents within the glycerophospholipids class. These changes were previously observed in our FAN model study [27].

Levels of the carnitine, acyl-carnitines, fatty acids, and fatty esters detected are significantly decreased in the fibrosis group compared to the control condition, except for palmitoyl-carnitine, the levels of which increased (Table S4 and Figure 4). The decrease in the levels of these compounds and the accumulation of palmitoyl-carnitine suggests a deficiency in the pathways that catabolize FFAs to produce energy and other intermediates in renal fibrosis [87]. This impairment in FAO could be driven by several converging signaling mechanisms associated with renal fibrosis. These may include the following: (1) downregulation of PPARα, a key transcriptional regulator of mitochondrial and peroxisomal FAO [88]; (2) suppression of AMPK signaling, which reduces CPT1 activity and mitochondrial lipid utilization [89]; (3) activation of the TGF-β/Smad3 pathway, which transcriptionally represses FAO genes [87]; (4) overactivation of the mTOR pathway, which shifts energy metabolism from oxidative phosphorylation toward glycolysis [90]; and (5) stabilization of HIF-1α under hypoxic conditions, which further suppresses FAO-related gene expression [91]. Together, these signaling alterations may promote mitochondrial dysfunction, alter lipid levels, and contribute to the progression of renal fibrosis. These lipid alterations, particularly the reduced levels of FFAs and acyl-carnitines, have also been observed in the plasma of patients with CKD, in which lower levels of these compounds correlated with higher GFR values [35,92]. Blockage of the β-oxidation process due to mitochondrial impairment would prevent the consumption of palmitoyl-carnitine for energy production [93]. Consequently, de novo biosynthesis of FFAs is not counteracting lipotoxic accumulation [94].

The levels of many different glycerophospholipids are altered due to kidney damage (Table S4 and Figure 4). In the glycerophosphatidylcholine class, we can observe a significant general decrease in lysophosphatidylcholines (LPC) and phosphatidylcholines (PC) in the WTOBS group compared to WTCT one. However, within this lipid class, the ether lipids from LPC and PC, with the alkyl ether substituent (“PC (O-)”) or alkenyl ether (“PC (P-)”, i.e., plasmalogens), follow a different trend to that of other glycerophosphocholines, with increased levels in the presence of fibrosis. The lysophosphatidylethanolamines (LPE), phosphatidylethanolamines (PE), phosphatidylglycerols (PG), phosphatidylserines (PS) and phosphatidylinositols (PI) follow the same trends as that of the glycerophosphocholine class. Alterations in phospholipid levels concerning kidney damage, renal fibrosis and CKD have been extensively evaluated in several studies [95]. However, the results vary according to the biological model used, the stage of the disease and the type of biological sample [96], and the molecular mechanism by which these ether lipids cause inflammation is not fully understood.

Within the glycerolipid category, levels of MG and DG were significantly decreased in the WTOBS group compared to the WTCT one (Table S4 and Figure 4). Our results for FA, MG and DG suggest that renal fibrosis induces metabolic changes in renal metabolism that lead to the degradation and consumption of complex lipids such as DGs, while preventing their synthesis using FFAs in other pathways.

Table S4 and Figure 4 show that, within the sphingolipid category, there was a significant decrease in ceramide (Cer) and sphingomyelin (SM) levels in the fibrosis group compared to the control. On the other hand, with regards to the use of glycosphingolipids, a general increase in hexosylceramides (HexCer) can be observed in WTOBS group. Sphingolipids have been recognized as essential lipids in the regulation of critical cellular functions [97]. The levels of the ceramides and hexosylceramides are increased in several kidney disease models, contributing to the signaling of different pathways involved in the progression of kidney injury through the regulation of apoptosis, inflammation and TGF-β signaling [98]. Based on our UUO7d dataset, we can see that the accumulation of hexosylceramides is likely to mediate the progression of fibrosis and renal damage. The reason for the decreased levels of ceramides and sphingomyelins may be their consumption for the production of hexosylceramides in advanced stages of CKD. In addition, previous studies on mouse and human kidney tissue have also shown decreases in certain ceramides, leading to concerns about the development and progression of kidney damage. On the other hand, the murine model of 2,8-dihydroxyadenine (DHA)-induced nephropathy has shown activation of mTOR downstream signaling, increased fibrosis, and oxidative stress, as well as an accumulation of hexosylceramides in kidney tissue [99].

Within the category of sterol lipids, there are alterations caused by renal damage. In this fibrosis model, the cholesteryl ester (CE) levels show a significant increase of about four-fold in the fibrosis group compared to the control group (Table S4 and Figure 4). Cholesterol has vital cellular functions, and the cholesterol ester cycle is highly regulated [100]. In conditions of high free cholesterol (FC) concentrations, CE formation is promoted to avoid toxicity due to FC accumulation [101]. FC is esterified into CE by sterol O-acyltransferases (SOATs), also known as acyl-CoA:cholesterol acyltransferases (ACATs), using free fatty acids as substrates. While this process may serve as a protective mechanism to buffer excess FC, excessive CE accumulation can promote lipid overload in kidney cells, contributing to inflammation, extracellular matrix deposition, and fibrosis progression [101]. Furthermore, chronic renal failure has been associated with upregulation of hepatic ACAT-2 expression, which may further enhance systemic CE levels [102]. These findings suggest that the increase in CE levels observed in our fibrosis model may reflect not only an adaptive response, but also a potentially maladaptive mechanism involved in the progression of kidney damage [100]. Overall, it appears that the progression of renal damage and the presence of fibrosis promotes an alteration in the cholesterol esterification cycle, inducing cellular membrane damage and CE accumulation.

In summary, the interstitial fibrosis associated with UUO dramatically impacts the renal lipidomic profile, altering the levels of several lipid classes. However, understanding how lipids are dysregulated in CKD is challenging owing to the incredible diversity of lipid structures, the alterations in their synthesis and catabolism, the interconnection between lipid families and the different modulation of changes linked to different cells and subcellular compartments [103].

## 3. Materials and Methods

### 3.1. Study Design

Mice were housed in a specific pathogen-free animal facility at CBMSO, adhering to EU regulations [104]. Following the 3 Rs (Replacement, Reduction, Refinement), we selected the minimal number of animals required to achieve meaningful results, avoiding unnecessary use of animals. We performed a power analysis based on the expected effect size and variability from prior studies [27,48]. A sample size of 8 mice per group achieves at least 80% power to detect a biologically meaningful difference at a significance level of 0.05. Therefore, this group size allowed for a feasible and ethical balance to obtain statistically relevant data.

**Figure 5:**
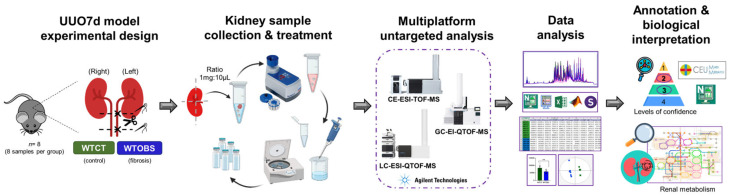
Study workflow. Experimental design of UUO7d murine model: kidney collection and sample treatment, untargeted metabolomics based on a multiplatform approach, data processing and analysis, compound annotation and biological interpretation. The figure was fully assembled using Microsoft PowerPoint for Microsoft 365, version 2405, with vector elements obtained from a free, royalty-free PNG image repository.

The UUO7d was established using a surgical procedure described in previous studies [33,105]. Briefly, mice were anesthetized with isoflurane, and the left ureter was doubly ligated with subsequent cutting between the ligatures. Adequate analgesia was provided to these animals after surgery, and the mice were sacrificed after seven days via CO_2_ overdose. Control and obstructed kidneys were harvested following perfusion with PBS. Kidney samples were categorized into two experimental groups (Figure 5), the wild-type control (WTCT) and wild-type obstruction (WTOBS) groups, each containing 8 samples.

### 3.2. Multiplatform Untargeted Metabolomic Analysis

#### 3.2.1. Reagents and Chemicals

All analytical-grade organic solvents and chemicals utilized in this study were obtained from Merck/Sigma-Aldrich (Darmstadt, Germany), VWR International/BDH Prolabo (Llinars del Valles, Barcelona, Spain) and Agilent Technologies (Santa Clara, CA, USA). Sialylation-grade pyridine was procured from VWR International BDH Prolabo. Reference mass solutions for LC-MS and CE-MS were obtained from Agilent Technologies. Deionized water (Milli-Q) utilized throughout the study was obtained using a Milli-Q PLUS system from Millipore (Vienna, Austria).

#### 3.2.2. Sample Treatment

Each kidney was dissected both lengthwise and crosswise to obtain four equal pieces. These sections were promptly frozen in liquid nitrogen and stored at −80 °C until further analysis.

For tissue disruption and homogenization, we followed our previously established protocol [17], briefly summarized in the Supplemental Materials. For each analytical platform, a different extraction method was applied [17]. Due to the limited availability of samples, GC-MS analysis was performed with only 6 samples of each group.

Quality control (QC) samples were prepared for each analytical technique by pooling equal volumes of each homogenized kidney tissue. Blank samples, which lacked the analyte of interest but underwent the same extraction protocols, were also prepared alongside the experimental samples. These blank samples were analyzed at the beginning and at the end of the analytical sequence. QC were injected at the beginning of the sequence to condition and stabilize the analysis system. Subsequently, they were injected after every 5–6 experimental samples throughout the run to assess the stability and performance of the analysis. Finally, they were also injected at the end of the analysis sequence. All experimental samples were randomized prior to sample preparation and analysis.

#### 3.2.3. Multiplatform Untargeted Metabolomic Profiling Based on CE-TOF-MS, UHPLC-QTOF-MS and GC-QTOF-MS Analyses

A multiplatform untargeted metabolomic analysis was performed using different Agilent Technologies platforms, such as an Agilent 7100 capillary electrophoresis system coupled with a 6224 TOF Mass Spectrometer, an Agilent Technologies 1290 Infinity II UHPLC system coupled with a 6545-quadrupole time-of-flight (QTOF) mass spectrometer and an Agilent 7890B GC instrument coupled with a 7250 QTOF mass spectrometer system. The analysis conditions and parameters were consistent with those described earlier [17] and are further explained in the Supplementary Materials.

### 3.3. Data Analysis

#### 3.3.1. Quality Control and Quality Assurance

The implementation of quality control and quality assurance procedures was carried out following the established guidelines [106]. MassHunter Qualitative analysis software (B.10.00, Agilent Technologies) was utilized to assess the quality of the acquired raw data. Furthermore, the consistency of the various ISs used in the analyses was examined at this stage. Subsequently, following the platform-specific data pre-processing step, the resulting raw data matrix was subjected to PCA-X using SIMCA P+16 (Umetrics^®^, Umea, Sweden) software [17]. This analysis aimed to observe signal drifts and variations in QC samples, and to identify potential outliers.

#### 3.3.2. Data Pre-Processing

For GC-MS data, spectral deconvolution and metabolite identification processes were carried out using MassHunter Workstation GC-MS Translator (B.04.01, Agilent Technologies) and MassHunter Unknown Analysis Tool 9.0 (Agilent Technologies) [107]. On the other hand, LC-MS and CE-MS data underwent pre-processing using Agilent MassHunter Profinder Software (B.08.00 and B.10.00, Agilent Technologies), following established steps described in previous publications [17,108]. For the CE-MS and LC-MS data, the software used for the pre-processing step was Agilent MassHunter Profinder (B.08.00 and B.10.00, Agilent Technologies), applying parameters and steps that have also been previously published [17,108].

#### 3.3.3. Data Pre-Treatment and Statistics

The data matrices obtained were imported into Microsoft Excel (Microsoft Office 2016), and the following steps were performed for data processing: blank subtraction, matrix curation, exclusion of metabolic features detected in less than 50% of QC, imputation of missing values based on the k-nearest neighbors method and normalization using biological model-driven normalization strategies [17,27]. The matrices were further filtered based on the coefficient of variation (CV) calculated from QC samples. Features with a CV below 20% in LC-MS and CE-MS matrices and below 30% in GC-MS data were retained.

For multivariate statistics analysis (MVA), unsupervised PCA-X models and supervised PLS-DA models were employed using SIMCA P+16 (Umetrics^®^, Umea, Sweden) software. Univariate statistical analysis (UVA) was performed using MATLAB R2018a (Mathworks, Inc., Natick, USA) software. A Mann–Whitney *U* test was applied to identify significant signals (*p* value < 0.05) by comparing the two groups (obstruction vs. control). The false discovery rate (FDR) was estimated using the standard Benjamini–Hochberg method (level α = 0.05) for multiple-hypothesis testing. The percentage of change (%Change) and the logarithm in base two of the fold change (Log2FC) were calculated for a comparison of the obstruction vs. control group.

#### 3.3.4. Metabolite Annotation

In the case of LC-MS and CE-MS, all statistically significant signals were annotated by searching for the accurate m/z values of the metabolic features in online databases using the advanced CEU Mass Mediator (CMM) tool [109]. The matched compounds were assigned based on accurate mass, with a maximum mass error of 20 ppm, considering isotopic pattern distribution, the potential formation of cations, adduct formation and the retention or migration time based on the separation conditions. In the case of CE-MS data, a range of different voltages were applied during the analysis to capture in-source ion fragmentation patterns characteristic of the various metabolites. This ion fragmentation information, along with the relative migration time (RMT) obtained by dividing the migration time of a feature by the migration time of the IS methionine sulfone, was compared to that in the CEMBIO in-house CE-MS database available in the CMM tool [110]. This comparison aimed at achieving a putative identification of the CE-MS metabolites (level 2).

## 4. Conclusions

In this study, employing a comprehensive multiplatform untargeted metabolomics approach, we meticulously examined the lipidomic and metabolic profiles of renal tissue under the UUO7d murine model. Leveraging three distinct separation techniques coupled with mass spectrometry, our methodology successfully identified a diverse array of compounds with varying physicochemical properties. This extensive evaluation has shed light on the profound metabolic alterations induced by tubulointerstitial fibrosis, specifically those associated with the UUO7d model, participating in numerous crucial biochemical pathways. Notably, this approach has allowed us to determine the alterations in the lipid profile of the UUO7d model, which had not been extensively evaluated previously. This multiplatform approach has underscored the pivotal role of assessing renal tissue in the study of CKD-associated fibrosis, providing a comprehensive depiction and metabolic map of the entire affected kidney in the widely utilized UUO model.

The use of multiplatform untargeted metabolomics was essential to reveal both broad and specific metabolic alterations in the UUO model of renal fibrosis. Each platform contributed uniquely to the overall map of affected pathways, allowing us to capture the complexity of biochemical dysregulation associated with fibrosis. This study not only demonstrates the value of combining multiple separation techniques but also provides a comprehensive and integrative metabolic fingerprint of fibrotic kidney tissue that could guide future research into potential therapeutic targets and early biomarkers.

Despite CKD’s pervasive global impact on public health, a thorough understanding of the intricate mechanisms driving its progression remains elusive, given the involvement of interconnected processes. This work supports the nexus between metabolism and renal fibrosis as a critical focal point to understand the development of this disease. This finding would help future endeavors dedicated to unraveling the molecular intricacies underpinning CKD and the identification of early biomarkers, aiming for substantial advancements in its management.

The findings from this study have significant translational implications for the understanding and treatment of CKD. By employing an untargeted multiplatform metabolomic approach, we identified key metabolic alterations associated with tubulointerstitial fibrosis in the UUO rodent model. These insights into disrupted metabolic pathways provide a deeper understanding of the molecular mechanisms driving CKD progression. Besides the insights into those alterations that we have gained, our results show that some of them could have potential as biomarkers of the alterations related to CKD and/or fibrosis. Some of them have been previously proposed as biomarkers as well, such as TMAO [111], citric acid [112], SAM [46], N-methyl-adenosine [113], N1-methyl-nicotinamide [114] and the group of HexCer [99]. To our knowledge, this is the first time that N1-acetylspermidine, 4-hydroxy-proline, 3-methyl-histidine, methyl-guanosine, and cholesteryl esters have been shown as potential biomarkers of such complications, and this could be further validated in a clinical context.

Our comprehensive metabolic profiling paves the way for the development of novel diagnostic tools and targeted therapies aimed at mitigating fibrosis and preserving renal function in CKD patients. Ultimately, these advancements could lead to improved clinical outcomes and quality of life for individuals affected by CKD.

It is important to acknowledge certain limitations inherent to our approach. Firstly, it is crucial to recognize that the animal models utilized for studying chronic kidney disease represent extreme conditions, and thus, extrapolating our findings directly to human scenarios requires further investigation. Additionally, while untargeted metabolomics proves to be a potent tool for hypothesis generation, it is imperative to conduct targeted studies to validate identified biomarkers and corroborate findings with other omics approaches. It is worth noting that, although our multiplatform approach allowed for comprehensive metabolic and lipidomic profiling of whole-kidney tissue, it lacked spatial resolution. The kidney is composed of distinct cell types and microenvironments, which are differentially affected by fibrosis. Thus, future studies applying spatial metabolomics could complement our findings by providing cell type- or region-specific insights into the metabolic reprogramming induced by UUO. Considering these in future studies will ensure the reliability and translational relevance of these findings.

## Data Availability

The datasets generated (primary MS data) and/or analyzed (feature areas) during the current study will be openly available in the Metabolomics Workbench repository at http://dx.doi.org/10.21228/M87N8N.

## Supplementary Materials

The following supporting information can be downloaded at https://www.mdpi.com/article/10.3390/ijms26104933/s1: References [17,27,110,115,116] are cited in supplemental materials.
