# Supplementary material for: Multiplatform Metabolomic Profiling of the Unilateral Ureteral Obstruction Murine Model of CKD
Source: Int J Mol Sci. 2025 May 21;26(10):4933. doi: 10.3390/ijms26104933 (PMC12112560; doi:10.3390/ijms26104933)
Supplement: Supplementary file 1 [file ijms-26-04933-s001.zip › UUO7d_SupplementalData.pdf]

Supplementary Material

# Multiplatform Metabolomic Profiling of the Unilateral Ureteral Obstruction Murine Model of CKD


Paula Cuevas-Delgado[1], Verónica Miguel[2], Francisco J. Rupérez[1], Santiago Lamas[2], Coral Barbas[1]

[1] Centre for Metabolomics and Bioanalysis (CEMBIO), School of Pharmacy, Universidad San Pablo-CEU, CEU Universities, Urbanización Montepríncipe, Boadilla del Monte, Madrid 28660, Spain.
[2] Program of Physiological and Pathological Processes, Centro de Biología Molecular "Severo Ochoa" (CBMSO, CSIC-UAM), c. Nicolás Cabrera 1, Madrid, Spain.


## 1. Results and Discussion

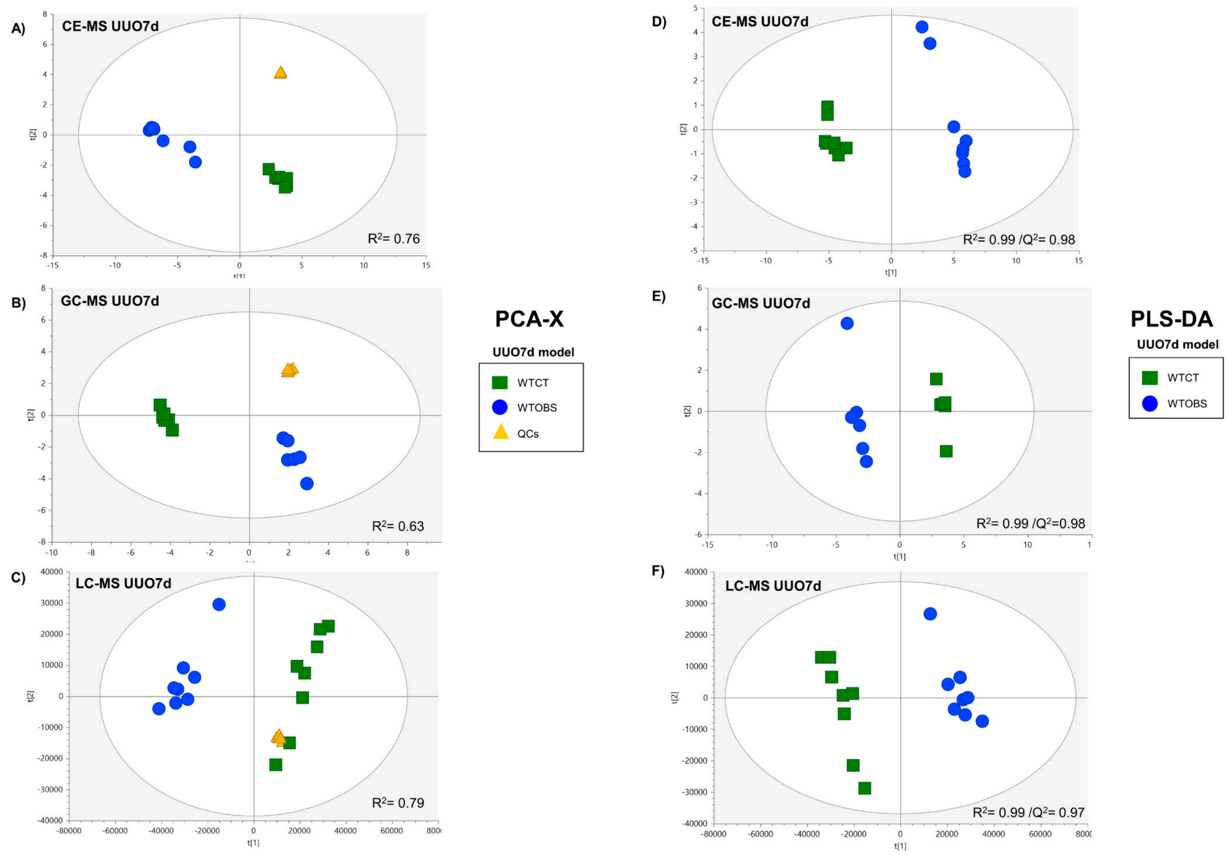


**Figure S1. PCA-X and PLS-DA models obtained with data from the three analytical platforms.** Both unsupervised and supervised models have been built on normalized data after applying its corresponding scaling and/or transformation methods. A), B) and C) PCA-X score plots for CE-MS, GC-MS and LC-MS data, respectively. **A)** CE-MS data with $Log_2$ transformation and Pareto scaling; a two-component model explains 55% ($R^2X$=0.549) and 21% ($R^2X$=0.208) variation in the 1st and the 2nd component $t_1$ and $t_2$, respectively **B)** GC-MS data with $Log_2$ transformation and Pareto scaling; a two-component model explains 40% ($R^2X$=0.399) and 23% ($R^2X$=0.225) variation in the 1st and the 2nd component $t_1$ and $t_2$, respectively **C)** LC-MS data with $Log_2$ transformation Unit-variance (UV) scaling, a two-component model explains 59% ($R^2X$=0.591) and 20% ($R^2X$=0.198) variation in the 1st and the 2nd component $t_1$ and $t_2$, respectively. D), E) and F) PLS-DA score plots for CE-MS, GC-MS and LC-MS data, respectively. **D)** PLS-DA CE-MS with

Pareto scale and Logarithm transformation ($R^2X$(cum) 0.731 $R^2Y$(cum) 0.994 $Q^2$(cum) 0.982); **E)** PLS-DA GC-MS with Pareto scale and Logarithm transformation ($R^2X$(cum) 0.621, $R^2Y$(cum) 0.997 $Q^2$(cum) 0.986); **F)** PLS-DA LC-MS with Unit-variance (UV) scale ($R^2X$(cum) 0.813, $R^2Y$(cum) 0.987 $Q^2$(cum) 0.972).

# 2. Materials and methods

## 2.2 Multiplatform Untargeted Metabolomics Analysis

### 2.2.2 Sample treatment

For tissue disruption and homogenization, a cold methanol solution of 50% was added to achieve a tissue-weight volume ratio of 1:10. Homogenization was conducted using a TissueLyser LT bead-mill homogenizer device (QIAGEN, Hilden, Germany) with 2.8 mm (mean diameter) steel beads [1]. The homogenization process entailed vibrating at a maximum power of 50Hz for 5 minutes, carried out over 4 cycles, with a 1-minute interval on ice between each cycle. The weight range of the kidney tissue varied between 25 and 50 mg (WTCT, 32–52.8 mg; WTOBS, 27.4–50.5 mg). Once the homogenate was obtained, the metabolite extraction process followed different protocols depending on the analytical platform used [2,3]. For CE-MS analysis, 100 µL of 0.2M formic acid was added to 100 µL of homogenate, followed by vortex-mixing and centrifugation (16,000×g for 10 minutes at 4 °C). The resulting supernatant was then transferred to a Centrifree ultracentrifugation device (Milipore Ireland Ltd., Cork, Ireland) equipped with a 30-kDa protein cutoff filter for deproteinization through centrifugation (2000×g for 70 minutes at 4 °C). The filtrate obtained was transferred to a chromacol vial and subsequently evaporated using a SpeedVac Concentrator (Thermo Fisher Scientific, Waltham, MA, USA). Finally, the residue was resuspended in 50 µL of 0.1 M formic acid containing 0.2 mM methionine sulfone as an internal standard (IS).

In the case of LC-MS and GC-MS analysis, 320 µL of cold methanol was added to 100 µL of homogenate, followed by the addition of 80 µL of methyl tert-butyl ether. The mixture was vortex-mixed for 1 hour at room temperature. Subsequently, the samples were centrifuged at 4000 g at 20ºC for 20 minutes, and the resulting supernatants were divided for LC-MS and GC-MS analysis. For LC-MS analysis, 100 µL of the supernatant was directly injected into the UHPLC-MS system. Regarding GC-MS analysis, 300 µL of the supernatant was transferred to a chromacol vial and evaporated to dryness using a SpeedVac Concentrator system. The derivatization process commenced by reconstituting the samples with 20 µL of O-methoxyamine hydrochloride (15 ng/mL) in pyridine for the methoximation step. After vigorous ultrasonication and vortex-mixing, the vials were incubated in the dark at room temperature for 16 hours. Subsequently, for the silylation step, 20 µL of BSTFA:TMCS (99:1) was added, followed by vortex-mixing for 5 minutes and incubation at 70ºC in the oven for 1 hour. Finally, 100 µL of heptane containing 20 ppm of tricosane (IS) was added. It is worth mentioning that due to the limited availability of samples, the GC-MS analysis was performed with only 6 samples in each group.

### 2.2.3 Multiplatform untargeted metabolomics profiling based on CE-TOF-MS, UHPLC-QTOF-MS and GC-QTOF-MS analyses

To conduct the CE-MS analysis, an Agilent Technologies 7100 capillary electrophoresis system coupled with a 6224 TOF Mass Spectrometer (Agilent Technologies) was utilized. The system was equipped with an electrospray ionization source (ESI). The analysis conditions have been previously documented [4]. In brief, a fused-silica capillary (Agilent Technologies; total length 96 cm; i.d.; 50 µm), previously flushed for 5 min with background electrolyte (BGE) (1 M formic acid in 10% methanol solution), was used for the separation analysis. Samples were injected over 50s

at 50mbar, and BGE was co-injected for 20s at 100mbar after each sample injection to improve the reproducibility of the analysis. The composition of the sheath liquid included methanol/water (1/1, v/v), formic acid (1 mM), and two reference masses (purine, m/z 121.050873; HP-0921, m/z 922.009798). The total time of the analytical run was 23 min. The separation was carried out at a pressure of 25 mbar and a voltage of +30kV, in positive ionization mode, with a flow rate of 0.6 mL/min and a split set to 1/100. The MS was operated in positive polarity mode, with a full scan range from m/z 70 to 1000 at a rate of 1.36 scan/s. The drying gas was set to 10 L/min at 200 ºC temperature, nebulizer to 10 psi, voltage to 3.5 kV, fragmentor to 125 V and skimmer to 65 V [4]. Towards the end of the sequence, two distinct methods were employed to obtain the in-source fragmentation (ISF) pattern of metabolites. These methods involved a fragmentor voltage of 200 V and a voltage range of 125-200 V. The ISF pattern was evaluated in a QC sample as well as in two samples from each experimental group [5].

To perform LC-MS metabolomics analysis, an Agilent Technologies 1290 Infinity II UHPLC system was employed, coupled with a 6545-quadrupole time-of-flight (QTOF) mass spectrometer (Agilent Technologies). The analysis encompassed both ESI+ (positive) and ESI- (negative) modes, enabling the detection of a wide range of metabolite ions. The analysis conditions were consistent with those previously described [3], 1.5 µL of samples were injected with a multiwash option using the Agilent 1290 Infinity II Multisampler system with a sample temperature of 15ºC. Reversed-phase chromatography was used with an InfinityLab Poroshell 120 EC-C8 (2.1 x 150 mm, 2.7 µm) column and a suitable guard column (Agilent Technologies) at 60ºC. Mobile phases for the positive ionization mode were composed by, for aqueous phase (solvent A), 10 mM ammonium formate in Milli-Q water and for organic phase (solvent B), 10mM ammonium formate in methanol/isopropanol (85/15, v/v) with a flow rate of 0.5 mL/min. The mobile phases gradient started at 75% of solvent B, increasing to 96% B at minute 23 and kept for 8 min. The gradient then increased to 100% of solvent B by minute 31.5 and was maintained until minute 32.5. At minute 33, the initial condition was returned, followed by a 7 min-re-equilibration time, with a total run time of 40 min. The parameters set in the mass spectrometer for the analysis in the positive and negative mode were as follows: 3500 V capillary voltage, 175 V fragmentor, 65 V skimmer, 750 V octupole radio frequency voltage, 11 L/min for drying gas flow rate at 290 ºC gas temperature, and 40 psi nebulizer pressure, 11L/min sheath gas flow, and 370ºC sheath gas temperature. The MS operated in full scan mode from 100 to 1700 m/z. The reference masses used over the whole analysis were m/z 121.0509 (protonated purine), m/z 149.0233 (protonated phtalic anhydride), and m/z 922.0098 (protonated HP-921) for positive ionization mode. An automated Calibrant Delivery System (CDS) constantly infused these reference masses at a 0.8 mL/min flow rate to allow constant mass correction, using a Dual Agilent Jet Stream Electrospray Ionization (Dual AJS ESI) source for continuously introducing calibrant solution.

The GC-MS analysis was carried out utilizing an Agilent 7890B GC instrument coupled with a 7250 QTOF mass spectrometer system (Agilent Technologies). The analysis conditions were consistent with those described earlier [2]. In summary, 1 µL of the derivatized sample was injected through an Agilent DB5-MS GC Capillary Column (30 m length, 0.25 mm, 0.25 µm film 95% dimethylpolysiloxane/5% diphenylpolysiloxane) using an Agilent autosampler (7693A). The samples were injected in a split ratio of 1:10 into a Restek 20782 deactivated glass-wool split liner. The injector port was established at 250ºC, the flow rate of helium carrier gas was set at 1 mL/min through the column. The temperature gradient was programmed at 60ºC as initial oven temperature (maintained for 1 min), increasing to 325ºC at a rate of 10ºC per minute. This temperature was kept for 10 min before cooling down. The total time of the analysis run was 37.5

minutes. The parameters used in the Agilent 7250 QTOF mass spectrometer system were: an electron ionization (EI) source set at 70 eV EI energy, 200ºC in the filament source temperature, 280ºC in the detector transfer line temperature, and 150ºC as quadrupole temperature. Finally, the mass spectrometer collected mass spectra data within a 40-600 m/z range at a scan rate of 10 spectra/s [2]

# 3. References

17. Cuevas-Delgado, P.; Dudzik, D.; Miguel, V.; Lamas, S.; Barbas, C. Data-dependent normalization strategies for untargeted metabolomics—a case study. *Anal. Bioanal. Chem.* **2020**, *412*, 6391–6405, https://doi.org/10.1007/s00216-020-02594-9.
27. Cuevas-Delgado, P.; Miguel, V.; Rupérez, F.J.; Lamas, S.; Barbas, C. Impact of renal tubular Cpt1a overexpression on the kidney metabolome in the folic acid-induced fibrosis mouse model. *Front. Mol. Biosci.* **2023**, *10*, 1161036, https://doi.org/10.3389/fmolb.2023.1161036.
110. Mamani-Huanca, M.; Gil de la Fuente, A.; Otero, A.; Gradillas, A.; Godzien, J.; Barbas, C.; López-Gonzálvez, Á. Enhancing confidence of metabolite annotation in Capillary Electrophoresis-Mass Spectrometry untargeted metabolomics with relative migration time and in-source fragmentation. *J. Chromatogr. A* **2021**, *1635*, 461758, https://doi.org/10.1016/j.chroma.2020.461758.
115. Fernández-García, M.; Rey-Stolle, F.; Boccard, J.; Reddy, V.P.; García, A.; Cumming, B.M.; Steyn, A.J.C.; Rudaz, S.; Barbas, C. Comprehensive Examination of the Mouse Lung Metabolome Following *Mycobacterium tuberculosis* Infection Using a Multiplatform Mass Spectrometry Approach. *J. Proteome Res.* **2020**, *19*, 2053–2070, https://doi.org/10.1021/acs.jproteome.9b00868.
116. Gonzalez-Riano, C.; Tapia-González, S.; Perea, G.; González-Arias, C.; DeFelipe, J.; Barbas, C. Metabolic Changes in Brain Slices over Time: a Multiplatform Metabolomics Approach. *Mol. Neurobiol.* **2021**, *58*, 3224–3237, https://doi.org/10.1007/s12035-020-02264-y.
