# Supplementary material for: Multiplatform Metabolomic Profiling of the Unilateral Ureteral Obstruction Murine Model of CKD
Source: Int J Mol Sci. 2025 May 21;26(10):4933. doi: 10.3390/ijms26104933 (PMC12112560; doi:10.3390/ijms26104933)
Supplement: Supplementary file 1 [file ijms-26-04933-s001.zip › UUO7d_SupplementalTables.pdf]

Table S1. Annotated metabolites found as statistically significant in WTOBS vs WTCT comparison present in TCA cycle, Urea cycle, folates and methionine cycles, and TMAO pathways.

Compound	Mass	RT	RMT	Formula	Analytical Platform & ESI mode	Mass Error	ID Source	ID Level	QCs CV (%)	WTOBS vs WTCT		
										%Change	Log_2FC	FDR
TCA cycle												
3-hydroxybutyric acid	104.047	8.17	--	C4H8O3	GC-MS	--	N	2	6	-39.8	-0.7	**0.015**
Citric acid	192.027	16.4	--	C6H8O7	GC-MS	--	F	2	11	2427.2	4.7	**0.025**
Succinic acid	118.027	10.5	--	C4H6O4	GC-MS	--	F	2	7	246.7	1.8	**0.002**
Fumaric acid	116.011	10.8	--	C4H4O4	GC-MS	--	F	2	27	224.2	1.7	**0.001**
Malic acid	134.021	12.6	--	C4H6O5	GC-MS	--	F	2	7	243.9	1.8	**0.004**
Urea cycle												
Argininosuccinic acid	272.113	7.69	0.8	C10H16N4O5	CE-MS (+)	2	ISF	2	2	35.4	0.4	**0.008**
Arginine	174.112	6.86	0.7	C6H14N4O2	CE-MS (+)	0	ISF	2	1	-31.7	-0.6	**0.001**
Guanidinoacetate	117.054	7.55	0.8	C3H7N3O2	CE-MS (+)	5	ISF	2	0	-78.4	-2.2	**$3.80x10^{-05}$**
Folate cycle & Methionine cycle												
Methionine	149.053	8.99	0.9	C5H11NO2S	CE-MS (+)	0	ISF	2	1	-26.6	-0.4	**$1.63x10^{-04}$**
S-adenosyl-methionine (SAM)	398.138	6.88	0.7	C15H23N6O5S	CE-MS (+)	2	ISF	2	2	115.4	1.1	**$2.80x10^{-04}$**
S-adenosyl-homocysteine (SAH)	384.123	7.92	0.8	C14H20N6O5S	CE-MS (+)	3	ISF	2	0	-44.3	-0.8	**$3.86x10^{-04}$**
Cysteine	121.02	9.28	1	C3H7NO2S	CE-MS (+)	4	ISF	2	1	64.6	0.7	**0.04**
N,N-dimethyl- glycine (DMG)	103.062	9.17	1	C4H9NO2	CE-MS (+)	15	ISF	2	1	-40.1	-0.7	**0.014**
N,N,N-trimethyl-glycine (Betaine)	117.079	9.38	1	C5H12NO2	CE-MS (+)	0	ISF	2	2	-44.8	-0.9	**$2.31x10^{-04}$**
Choline	104.107	6.56	0.7	C5H14NO	CE-MS (+)	2	ISF	2	1	-16.2	-0.3	**0.012**
TMAO												
Trimethlyamine N-oxide (TMAO)	75.0688	6.38	0.7	C3H9NO	CE-MS (+)	5	ISF	3	2	177.2	1.5	**0.016**

Compound names after annotation process (compound), monoisotopic mass (mass), retention time or migration time expressed in minutes (RT), relative migration time for CE-MS compounds based on the migration time of the internal standard (RMT), chemical formula, analytical platform and ESI mode used for the detection of the compound: LC-MS, GC-MS or CE-MS, adduct with which the compound is detected, mass error expressed in PPM (error), ID source (ID S): DB: Database, F: Fiehn Library, ISF: in-source fragmentation pattern, LA: Lipid Annotator (MS/MS), N: Nist Library. ID levels (ID L): 1 standard, 2 putative, 3 tentative, 4 molecular formula, 5 Unknown, based on Schrimpe-Rutledge A. et al classification (1). Coefficient of variation of the metabolite in QC samples (QCs CV). %Change in the specific comparison obtained as Cases vs. Controls; logarithm in base two of the fold change obtained as Cases vs. Controls (Log_2FC), consequently in the %Change and $Log_2FC$the negative sign (-) and blue colour indicates that the metabolite is less abundant in the case group than in the control group, and the positive sign (+) and red colour means that the metabolites is more abundant in the case group than in control group, and *p* value obtained with Benjamini-Hochberg correction performing false discovery rate (FDR).

Table S2. Annotated metabolites found as statistically significant in WTOBS vs WTCT comparison present in modified amino acids (MAAs), polyamines, and amino acids metabolism.

Compound	Mass	RT	RMT	Formula	Analytical Platform & ESI mode	Mass Error	ID Source	ID Level	QCs CV (%)	WTOBS vs WTCT		
										%Change	Log_2FC	FDR
Modified Amino acids (MAAs)												
N2-methyl-lysine	160.121	7.2	0.7	C7H16N2O2	CE-MS (+)	0	ISF	2	1	-53.5	-1.1	**0.014**
N6-methyl-lysine	160.121	6.83	0.7	C7H16N2O2	CE-MS (+)	0	ISF	2	1	-44	-0.8	**0.042**
N,N,N-trimethyl-lysine	188.152	6.88	0.7	C9H20N2O2	CE-MS (+)	4	ISF	2	2	36.7	0.5	**0.001**
5-hydroxy-lysine	162.101	6.89	0.7	C6H14N2O3	CE-MS (+)	3	ISF	2	1	-84.3	-2.7	**$2.80x10^{-04}$**
N2-Acetyl-lysine	188.116	8.06	0.8	C8H16N2O3	CE-MS (+)	0	ISF	2	2	-20.9	-0.3	**0.008**
N-methyl-alanine	103.064	8.4	0.9	C4H9NO2	CE-MS (+)	9	ISF	2	1	36.1	0.4	**0.001**
4-hydroxy-proline	131.058	9.27	1	C5H9NO3	CE-MS (+)	2	ISF	2	1	-80.4	-2.4	**0.001**
3-methyl-histidine	169.086	7.1	0.7	C7H11N3O2	CE-MS (+)	2	ISF	2	2	-81.8	-2.5	**$1.50x10^{-05}$**
5-hydroxy-tryptophan	220.087	10.2	1.1	C11H12N2O3	CE-MS (+)	9	ISF	2	1	-64.3	-1.5	**0.001**
N-acetyl-glutamic acid	189.064	16.1	--	C7H11NO5	GC-MS	--	F	2	11	497.6	2.6	**$6.70x10^{-05}$**

Polyamines												
Putrescine	88.1002	4.96	0.5	C4H12N2	CE-MS (+)	2	ISF	2	0	-68.1	-1.6	**$5.74x10^{-05}$**
Spermidine	145.158	4.83	0.5	C7H19N3	CE-MS (+)	0	ISF	2	1	92.1	0.9	**$1.49x10^{-05}$**
Spermine	202.217	4.8	0.5	C10H26N4	CE-MS (+)	4	ISF	2	0	-32.7	-0.6	**$2.62x10^{-05}$**
N1-acetylspermidine	187.168	6.25	0.7	C9H21N3O	CE-MS (+)	3	ISF	2	3	847.1	3.2	**$1.49x10^{-05}$**
Amino acids												
Alanine	89.048	8.05	0.8	C3H7NO2	CE-MS (+)	4	ISF	2	3	-28.8	-0.5	**$1.50x10^{-05}$**
Proline	115.063	8.91	0.9	C5H9NO2	CE-MS (+)	0	ISF	2	5	-45.7	-0.9	**0.001**
Valine	117.079	8.61	0.9	C5H11NO2	CE-MS (+)	1	ISF	2	1	-36	-0.6	**$1.49x10^{-05}$**
Isoleucine/Leucine	131.094	8.76	0.9	C6H13NO2	CE-MS (+)	2	ISF	3	1	-25.9	-0.4	**0.001**
Histidine	155.07	6.98	0.7	C6H9N3O2	CE-MS (+)	1	ISF	2	1	-30.5	-0.5	**$3.30x10^{-05}$**
Phenylalanine	165.079	9.18	1	C9H11NO2	CE-MS (+)	1	ISF	2	0	-13.6	-0.2	**0.001**
Tyrosine	181.074	9.33	1	C9H11NO3	CE-MS (+)	0	ISF	2	2	-29.2	-0.5	**$1.49x10^{-05}$**
Tryptophan	204.09	9.14	1	C11H12N2O2	CE-MS (+)	1	ISF	2	0	-20.1	-0.3	**$3.93x10^{-04}$**
Asparagine	132.052	8.87	0.9	C4H8N2O3	CE-MS (+)	10	ISF	2	1	-17.7	-0.3	**0.001**
Lysine	146.106	6.7	0.7	C6H14N2O2	CE-MS (+)	0	ISF	2	1	-32.8	-0.6	**0.014**
Serine	105.043	8.6	0.9	C3H7NO3	CE-MS (+)	2	ISF	2	0	-30.8	-0.5	**$2.62x10^{-05}$**
Glutamine	146.069	9.03	0.9	C5H10N2O3	CE-MS (+)	4	ISF	2	1	-23.4	-0.4	**0.004**
Aspartic acid	133.037	9.42	1	C4H7NO4	CE-MS (+)	1	ISF	2	0	-40.8	-0.8	**$3.51x10^{-05}$**
Cystine	240.024	9.23	1	C6H12N2O4S2	CE-MS (+)	0	ISF	2	1	23.4	0.3	**0.013**

Compound names after annotation process (compound), monoisotopic mass (mass), retention time or migration time expressed in minutes (RT), relative migration time for CE-MS compounds based on the migration time of the internal standard (RMT), chemical formula, analytical platform and ESI mode used for

the detection of the compound: LC-MS, GC-MS or CE-MS, adduct with which the compound is detected, mass error expressed in PPM (error), ID source (ID S): DB: Database, F: Fiehn Library, ISF: in-source fragmentation pattern, LA: Lipid Annotator (MS/MS), N: Nist Library. ID levels (ID L): 1 standard, 2 putative, 3 tentative, 4 molecular formula, 5 Unknown, based on Schrimpe-Rutledge A. et al classification (1). Coefficient of variation of the metabolite in QC samples (QCs CV). %Change in the specific comparison obtained as Cases vs. Controls; logarithm in base two of the fold change obtained as Cases vs. Controls (Log_2FC), consequently in the %Change and $Log_2FC$the negative sign (-) and blue colour indicates that the metabolite is less abundant in the case group than in the control group, and the positive sign (+) and red colour means that the metabolites is more abundant in the case group than in control group, and *p* value obtained with Benjamini-Hochberg correction performing false discovery rate (FDR).

Table S3. Annotated metabolites found as statistically significant in WTOBS vs WTCT comparison present in purines metabolism, vitamins, and cofactors.

Compound	Mass	RT	RMT	Formula	Analytical Platform & ESI mode	Mass Error	ID Source	ID Level	QCs CV (%)	WTOBS vs WTCT		
										%Change	Log_2FC	FDR
Purines metabolism												
Adenine	135.054	17	--	C14H28O2	GC-MS	--	F	2	7	146.6	1.3	**0.001**
Adenosine	267.0969	18.3	0.8	C10H13N5O4	CE-MS (+)	3	ISF	2	1.3	-60.2	-1.3	**0.006**
N-methyl-adenosine	281.1127	18.4	0.8	C11H15N5O4	CE-MS (+)	17	ISF	2	4.5	1191.4	3.7	**$4.66x10^{-05}$**
Inosine	268.0805	23.3	--	C10H12N4O5	GC-MS	--	F	2	3.9	-61.9	-1.4	**0.007**
Methyl-guanine	165.067	7.53	0.8	C6H7N5O	CE-MS (+)	14	ISF	2	1	247.9	1.8	**0.001**
Guanosine	283.091	10.2	1.1	C10H13N5O5	CE-MS (+)	4	ISF	2	1	-49.7	-1	**0.016**
Methyl-guanosine	297.107	9.84	1	C11H15N5O5	CE-MS (+)	2	ISF	2	1	WTOBS	WTOBS	--
Deoxyguanosine	267.094	10.4	1.1	C10H13N5O4	CE-MS (+)	11	ISF	2	1	-70.2	-1.7	**$8.68x10^{-05}$**
Vitamins and cofactors												
Nicotinamide (Niacinamide, vit B3)	122.048	12.6	--	C6H6N2O	GC-MS	--	F	2	7	-41.5	-0.8	**0.01**
N1-methyl-nicotinamide	136.063	6.92	0.7	C7H8N2O	CE-MS (+)	2	ISF	2	2	1209	3.7	**$5.94x10^{-05}$**
Pyridoxal (vit B6)	167.058	7.89	0.8	C8H9NO3	CE-MS (+)	3	ISF	2	2	-59.6	-1.3	**0.001**
Pyridoxamine (vit B6)	168.089	5.99	0.6	C8H12N2O2	CE-MS (+)	7	DB	3	1	-95.5	-4.5	**$3.30x10^{-05}$**

Pantothenate (vit B5)	219.11	13.5	1.4	C9H17NO5	GC-MS	--	F	2	20	-66.9	-1.6	**0.008**
Thiamine (vit B1)	265.112	6.45	0.7	C12H17N4OS	CE-MS (+)	1	ISF	2	2	-55.1	-1.2	**$1.50x10^{-05}$**
Dehydroascorbic acid (vit C)	174.016	16.7	--	C6H6O6	GC-MS	--	F	2	5	136.1	1.2	**0.002**

Compound names after annotation process (compound), monoisotopic mass (mass), retention time or migration time expressed in minutes (RT), relative migration time for CE-MS compounds based on the migration time of the internal standard (RMT), chemical formula, analytical platform and ESI mode used for the detection of the compound: LC-MS, GC-MS or CE-MS, adduct with which the compound is detected, mass error expressed in PPM (error), ID source (ID S): DB: Database, F: Fiehn Library, ISF: in-source fragmentation pattern, LA: Lipid Annotator (MS/MS), N: Nist Library. ID levels (ID L): 1 standard, 2 putative, 3 tentative, 4 molecular formula, 5 Unknown, based on Schrimpe-Rutledge A. et al classification (1). Coefficient of variation of the metabolite in QC samples (QCs CV). %Change in the specific comparison obtained as Cases vs. Controls; logarithm in base two of the fold change obtained as Cases vs. Controls (Log_2FC), consequently in the %Change and $Log_2FC$the negative sign (-) and blue colour indicates that the metabolite is less abundant in the case group than in the control group, and the positive sign (+) and red colour means that the metabolites is more abundant in the case group than in control group, and *p* value obtained with Benjamini-Hochberg correction performing false discovery rate (FDR).

Table S4. Annotated lipids found as statistically significant in WTOBS vs WTCT comparison ordered according to the different families and classes.

Compound	Mass	RT	RMT	Formula	Analytical Platform & ESI mode	Mass Error	ID Source	ID Level	QCs CV (%)	WTOBS vs WTCT		
										%Change	Log_2FC	FDR
Fatty acyls												
Carnitine and Acylcarnitines												
Carnitine	161.105	7.7	0.8	C7H15NO3	CE-MS (+)	3	ISF	2	2	-27.7	-0.5	**0.001**
Acetyl-carnitine (CAR 2:0)	203.116	8.03	0.8	C9H17NO4	CE-MS (+)	1	ISF	2	2	-38.4	-0.7	**0.005**
Palmitoylcarnitine (CAR 16:0)	399.336	2.34	--	C23H45NO4	LC-MS (+)	2	DB	3	2	119	1.1	**0.009**
Fatty acids and conjugates												
Isocaproic acid (FA 6:0)	116.084	0.71	--	C6H12O2	LC-MS (+)	0	DB	3	2	-57.7	-1.2	**0.006**
Suberic acid (FA 8:1;O2)	174.083	0.69	--	C8H14O4	LC-MS (+)	1	DB	3	5	-40.2	-0.7	**0.044**
Linoleic acid (FA 18:2)	280.2402	20.4	--	C18H32O2	GC-MS	--	F	2	5.1	-43.6	-0.8	**$4.99x10^{-04}$**
Fatty esters												

Methylpalmitate	270.256	17.6	--	C17H34O2	GC-MS	--	F	2	7	-50.4	-1	**0.001**
Methylarachidonate	318.257	5.37	--	C21H34O2	LC-MS (+)	2	DB	3	4	-46.9	-0.9	**$4.99x10^{-04}$**
Glycerophospholipids												
Glycerophosphocholines												
Monoacylglycerophosphocholines												
LPC P-18:0	507.3693	3.59	--	C26H54NO6P	LC-MS (+)	1	DB	3	3	24.5	0.3	**0.044**
LPC 22:6	567.3331	2.28	--	C30H50NO7P	LC-MS (+)	5	DB	3	15	-38.6	-0.7	**0.002**
Diacylglycerophosphocholines												
PC P-36:0 | PC O-36:1	773.6272	17.4	--	C44H88NO7P	LC-MS (+)	4	DB	3	6	86.4	0.9	**0.002**
PC P-36:3 | PC O-36:4	767.5845	14.7	--	C44H82NO7P	LC-MS (+)	2	DB	3	1	97.4	1	**$4.99x10^{-04}$**
PC P-36:4 | PC O-36:5	765.5645	14.5	--	C44H80NO7P	LC-MS (+)	4	DB	3	3	76.7	0.8	**$4.99x10^{-04}$**
PC P-38:4 | PC O-38:5	793.5969	16.3	--	C46H84NO7P	LC-MS (+)	2	DB	3	2	93.2	1	**$4.99x10^{-04}$**
PC 32:0	733.5639	14	--	C40H80NO8P	LC-MS (+)	2	DB	3	1	-31.1	-0.5	**$4.99x10^{-04}$**
PC 36:1	787.6102	16.3	--	C44H86NO8P	LC-MS (+)	1	DB	3	3	-32	-0.6	**$4.99x10^{-04}$**
PC 36:3	783.5786	14.1	--	C44H82NO8P	LC-MS (+)	0	DB	3	19	-40.9	-0.8	**0.003**
PC 37:2	799.6069	16.2	--	C45H86NO8P	LC-MS (+)	3	DB	3	2	-28.4	-0.5	**0.003**
PC 37:7	811.5141	15.4	--	C45H76NO8P	LC-MS (+)	2	DB	3	2	-49	-1	**0.001**
PC 38:2	835.6057	16.2	--	C46H88NO8P	LC-MS (+)	1	DB	3	3	-63.2	-1.4	**$4.99x10^{-04}$**
PC 38:3	811.6087	15.2	--	C46H86NO8P	LC-MS (+)	1	DB	3	2	-60	-1.3	**$4.99x10^{-04}$**
PC 38:3	811.6091	16	--	C46H86NO8P	LC-MS (+)	0	DB	3	3	-41.4	-0.8	**0.001**
PC 38:4	809.5944	15.3	--	C46H84NO8P	LC-MS (+)	1	DB	3	3	-29.8	-0.5	**0.004**

PC 38:5	807.5797	14.2	--	C46H82NO8P	LC-MS (+)	2	DB	3	1	-48.2	-1	**4.99x10^{-04}**
PC 39:4	823.6155	16.2	--	C47H86NO8P	LC-MS (+)	8	DB	3	3	-21.2	-0.3	**0.009**
PC 40:4	837.6247	17.1	--	C48H88NO8P	LC-MS (+)	0	DB	3	4	-33.3	-0.6	**4.99x10^{-04}**
PC 40:5	835.6058	15.8	--	C48H86NO8P	LC-MS (+)	4	DB	3	3	-60.5	-1.3	**4.99x10^{-04}**
PC 40:6	855.5685	15.2	--	C48H84NO8P	LC-MS (+)	8	DB	3	4	-42.1	-0.8	**0.013**
PC 40:7	815.5816	13.9	--	C48H82NO7P	LC-MS (+)	2	DB	3	14	-49.1	-1	**0.003**
PC 40:8	829.5613	12.9	--	C48H80NO8P	LC-MS (+)	1	DB	3	18	-54	-1.1	**4.99x10^{-04}**
PC 42:10	853.5583	12.8	--	C50H80NO8P	LC-MS (+)	5	DB	3	7	-53.7	-1.1	**4.99x10^{-04}**
Glycerophosphoethanolamines												
Monoacylglycerophosphoethanolamines												
LPE 18:0	481.3198	3.72	--	C23H48NO7P	LC-MS (+)	6	DB	3	6	-62.1	-1.4	**4.99x10^{-04}**
Diacylglycerophosphoethanolamines												
PE 34:1	717.5432	14.6	--	C39H76NO8P	LC-MS (+)	17	DB	3	2	-44.6	-0.9	**4.99x10^{-04}**
PE 34:2	715.5181	13.6	--	C39H74NO8P	LC-MS (+)	4	DB	3	2	-65.1	-1.5	**4.99x10^{-04}**
PE 36:2	743.5576	15.3	--	C41H78NO8P	LC-MS (+)	15	DB	3	2	-65.7	-1.5	**4.99x10^{-04}**
PE 36:3	741.5365	14.1	--	C41H76NO8P	LC-MS (+)	7	DB	3	4	-74.5	-2	**4.99x10^{-04}**
PE 36:4	739.5169	13.6	--	C41H74NO8P	LC-MS (+)	2	DB	3	2	-77.3	-2.1	**4.99x10^{-04}**
PE 37:4	753.535	14.5	--	C42H76NO8P	LC-MS (+)	5	DB	3	6	-65.4	-1.5	**4.99x10^{-04}**
PE 38:4	767.5497	15.4	--	C43H78NO8P	LC-MS (+)	4	DB	3	2	-72	-1.8	**8.77x10^{-04}**
PE 38:5	765.5356	14.2	--	C43H76NO8P	LC-MS (+)	6	DB	3	1	-80.9	-2.4	**4.99x10^{-04}**
PE 38:6	763.5183	13.5	--	C43H74NO8P	LC-MS (+)	4	DB	3	3	-66	-1.6	**4.99x10^{-04}**
PE 39:4	781.5634	13.6	--	C44H80NO8P	LC-MS (+)	1	DB	3	1	-39.6	-0.7	**4.99x10^{-04}**

PE 40:6	833.592	15.2	--	C48H84NO8P	LC-MS (+)	2	DB	3	2	-41.1	-0.8	**0.034**
Glycerophosphoglycerols												
Diacylglycerophosphoethanolamines												
PG 36:3	789.5448	14	--	C42H77O10P	LC-MS (+)	9	DB	3	1	-63.9	-1.5	**4.99x10^{-04}**
PG 38:2	819.6073	15.5	--	C44H83O10P	LC-MS (+)	10	DB	3	6	-29.5	-0.5	**0.034**
PG 38:4	895.5305	13.1	--	C44H80O13P2	LC-MS (+)	1	DB	3	19	-52.6	-1.1	**4.99x10^{-04}**
PG 38:4	895.531	13.4	--	C44H80O13P2	LC-MS (+)	3	DB	3	2	-46.7	-0.9	**0.013**
PG 40:4	923.5597	15.2	--	C46H84O13P2	LC-MS (+)	6	DB	3	1	-47.1	-0.9	**0.013**
PG 44:12	963.5166	13.4	--	C50H76O13P2	LC-MS (+)	15	DB	3	6	-47.3	-0.9	**0.009**
Glycerophosphoserines												
Diacylglycerophosphoserines												
PS 37:0	805.5903	15.4	--	C43H84NO10P	LC-MS (+)	9	DB	3	1	-43.1	-0.8	**4.99x10^{-04}**
PS 38:4	811.5352	14	--	C44H78NO10P	LC-MS (+)	1	DB	3	7	-51	-1	**4.99x10^{-04}**
PS 38:4	811.5312	13.6	--	C44H78NO10P	LC-MS (+)	6	DB	3	4	-34.2	-0.6	**4.99x10^{-04}**
PS 40:6	835.5366	13.5	--	C46H78NO10P	LC-MS (+)	0	DB	3	1	-21.7	-0.4	**0.001**
Glycerophosphoinositols												
Diacylglycerophosphoinositols												
PI 36:1	886.5544	13.2	--	C45H85O13P	LC-MS (+)	0	DB	3	3	-36.4	-0.7	**4.99x10^{-04}**
PI 38:7	897.544	14.2	--	C47H77O13P	LC-MS (+)	8	DB	3	4	-22.3	-0.4	**0.018**
Glycerolipids												
Monoradylglycerols												
Monoacylglyerols												
MG 16:0	352.2601	3.41	--	C19H38O4	LC-MS (+)	3	DB	3	9	-23.6	-0.4	**4.99x10^{-04}**
MG 18:0	380.2903	4.91	--	C21H42O4	LC-MS (+)	6	DB	3	5	-50	-1	**4.99x10^{-04}**

Diradylglycerols												
Diacylglyerols												
DG 32:0	590.4892	16.6	--	C35H68O5	LC-MS (+)	1	DB	3	4	-35.5	-0.6	**4.99x10^{-04}**
DG 34:0	618.5274	18.1	--	C37H72O5	LC-MS (+)	12	DB	3	4	-26.1	-0.4	**0.004**
DG 34:1	616.5048	17.1	--	C37H70O5	LC-MS (+)	1	DB	3	3	-45.8	-0.9	**4.99x10^{-04}**
DG 34:1	580.5436	18.3	--	C37H72O4	LC-MS (+)	5	DB	3	7	-57.7	-1.2	**4.99x10^{-04}**
DG 36:1	644.5383	17.7	--	C39H74O5	LC-MS (+)	4	DB	3	3	-33.2	-0.6	**0.044**
DG 36:1	644.5358	18.6	--	C39H74O5	LC-MS (+)	0	DB	3	8	-31.7	-0.5	**4.99x10^{-04}**
DG 34:2	614.4894	16.1	--	C37H68O5	LC-MS (+)	1	DB	3	3	-43.1	-0.8	**4.99x10^{-04}**
DG 36:2	642.521	17.6	--	C39H72O5	LC-MS (+)	2	DB	3	1	-23.2	-0.4	**0.034**
DG 36:3	640.5044	16.6	--	C39H70O5	LC-MS (+)	1	DB	3	7	-47.7	-0.9	**4.99x10^{-04}**
DG 36:4	638.4896	16.1	--	C39H68O5	LC-MS (+)	1	DB	3	3	-51	-1	**4.99x10^{-04}**
DG 38:3	668.5355	18.2	--	C41H74O5	LC-MS (+)	1	DB	3	2	-61.8	-1.4	**4.99x10^{-04}**
DG 38:4	668.5368	18.2	--	C43H72O5	LC-MS (+)	2	DB	3	5	-67	-1.6	**4.99x10^{-04}**
DG 38:5	664.4999	16.6	--	C41H70O5	LC-MS (+)	1	DB	3	3	-37.1	-0.7	**0.002**
DG 38:6	662.488	15.9	--	C41H68O5	LC-MS (+)	1	DB	3	2	-41.1	-0.8	**0.003**
DG 40:6	690.5181	17.5	--	C43H72O5	LC-MS (+)	3	DB	3	3	-48.3	-1	**0.006**
Sphingolipids												
Ceramides												
N-acylsphingosines (ceramides)												
Cer 40:0;O2	645.6039	17.7	--	C40H81NO3	LC-MS (+)	0	DB	3	1	-36.8	-0.7	**4.99x10^{-04}**
Cer 34:1;O2	537.5132	13.3	--	C34H67NO3	LC-MS (+)	2	DB	3	3	-33.8	-0.6	**0.006**
Cer 40:1;O2	621.6062	18.3	--	C40H79NO3	LC-MS (+)	0	DB	3	2	-34.2	-0.6	**0.001**
Cer 42:1;O2	649.6377	19.7	--	C42H83NO3	LC-MS (+)	1	DB	3	2	-33.1	-0.6	**0.001**
Cer 34:2;O2	535.497	12	--	C34H65NO3	LC-MS (+)	1	DB	3	1	-29.8	-0.5	**0.034**
Cer 40:2;O2	619.59	17.4	--	C40H77NO3	LC-MS (+)	1	DB	3	3	-48.2	-1	**0.004**
Cer 42:2;O2	647.6216	18.5	--	C42H81NO3	LC-MS (+)	0	DB	3	1	-29.1	-0.5	**4.99x10^{-04}**
Neutral glycosphingolipids												

Simple Glc series (Hexosylceramides)												
HexCer 34:1;O2	721.5457	12	--	C40H77NO8	LC-MS (+)	2	DB	3	7	118.1	1.1	**0.003**
HexCer 38:1;O2	771.6141	16.3	--	C44H86NO7P	LC-MS (+)	11	DB	3	2	57.3	0.7	**0.001**
HexCer 38:2;O2	831.6588	17.3	--	C48H91NO8	LC-MS (+)	3	DB	3	2	104.8	1	**0.001**
HexCer 42:2;O2	965.6766	15.5	--	C54H95NO13	LC-MS (+)	4	DB	3	3	254	1.8	**0.003**
Phosphosphingolipids												
Sphingomyelins												
SM 34:1;O2	702.569	12.2	--	C39H79N2O6P	LC-MS (+)	2	DB	3	2	-39.1	-0.7	**4.99x10^{-04}**
SM 38:1;O2	758.6284	15.8	--	C43H87N2O6P	LC-MS (+)	2	DB	3	2	-24.4	-0.4	**0.006**
SM 40:1;O2	786.662	17.5	--	C45H91N2O6P	LC-MS (+)	1	DB	3	1	-46.6	-0.9	**4.99x10^{-04}**
SM 41:1;O2	800.6762	18.3	--	C46H93N2O6P	LC-MS (+)	1	DB	3	2	-35.7	-0.6	**4.99x10^{-04}**
SM 42:1;O2	814.6931	19	--	C47H95N2O6P	LC-MS (+)	0	DB	3	1	-49.3	-1	**4.99x10^{-04}**
SM 34:2;O2	700.5521	11	--	C39H77N2O6P	LC-MS (+)	0	DB	3	3	-35.1	-0.6	**4.99x10^{-04}**
SM 40:2;O2	784.6447	16.5	--	C45H89N2O6P	LC-MS (+)	2	DB	3	2	-42.8	-0.8	**0.002**
SM 42:2;O2	812.6777	17.7	--	C47H93N2O6P	LC-MS (+)	1	DB	3	2	-34.5	-0.6	**4.99x10^{-04}**
SM 42:2;O2	812.6775	18.1	--	C47H93N2O6P	LC-MS (+)	0	DB	3	2	-60.2	-1.3	**4.99x10^{-04}**
Sterol Lipids												
Sterols												
Cholesterol and derivatives												
CE 18:2	686.5395	24.3	--	C45H76O2	LC-MS (+)	2	DB	3	4	162.6	1.4	**4.99x10^{-04}**
CE 20:4	710.5408	24	--	C47H76O2	LC-MS (+)	0	DB	3	2	200.3	1.6	**0.002**
CE 22:6	734.5415	23.8	--	C49H76O2	LC-MS (+)	1	DB	3	3	1928.6	4.3	**4.99x10^{-04}**

Compound names after annotation process (compound), monoisotopic mass (mass), retention time or migration time expressed in minutes (RT), relative migration time for CE-MS compounds based on the migration time of the internal standard (RMT), chemical formula, analytical platform and ESI mode used for

the detection of the compound: LC-MS, GC-MS or CE-MS, adduct with which the compound is detected, mass error expressed in PPM (error), ID source (ID S): DB: Database, F: Fiehn Library, ISF: in-source fragmentation pattern, LA: Lipid Annotator (MS/MS), N: Nist Library. ID levels (ID L): 1 standard, 2 putative, 3 tentative, 4 molecular formula, 5 Unknown, based on Schrimpe-Rutledge A. et al classification (1). Coefficient of variation of the metabolite in QC samples (QCs CV). %Change in the specific comparison obtained as Cases vs. Controls; logarithm in base two of the fold change obtained as Cases vs. Controls (Log_2FC), consequently in the %Change and $Log_2FC$the negative sign (-) and blue colour indicates that the metabolite is less abundant in the case group than in the control group, and the positive sign (+) and red colour means that the metabolites is more abundant in the case group than in control group, and *p* value obtained with Benjamini-Hochberg correction performing false discovery rate (FDR). LPC(P-XX:Y): Plasmalogen lysophosphatidylcholine; PC(P-XX:Y): Plasmalogen phosphatidylcholine. All other coding, as referred in text.
