# Supplementary figures and images for: Multiplatform Metabolomic Profiling of the Unilateral Ureteral Obstruction Murine Model of CKD
Source: Int J Mol Sci. 2025 May 21;26(10):4933. doi: 10.3390/ijms26104933 (PMC12112560; doi:10.3390/ijms26104933)

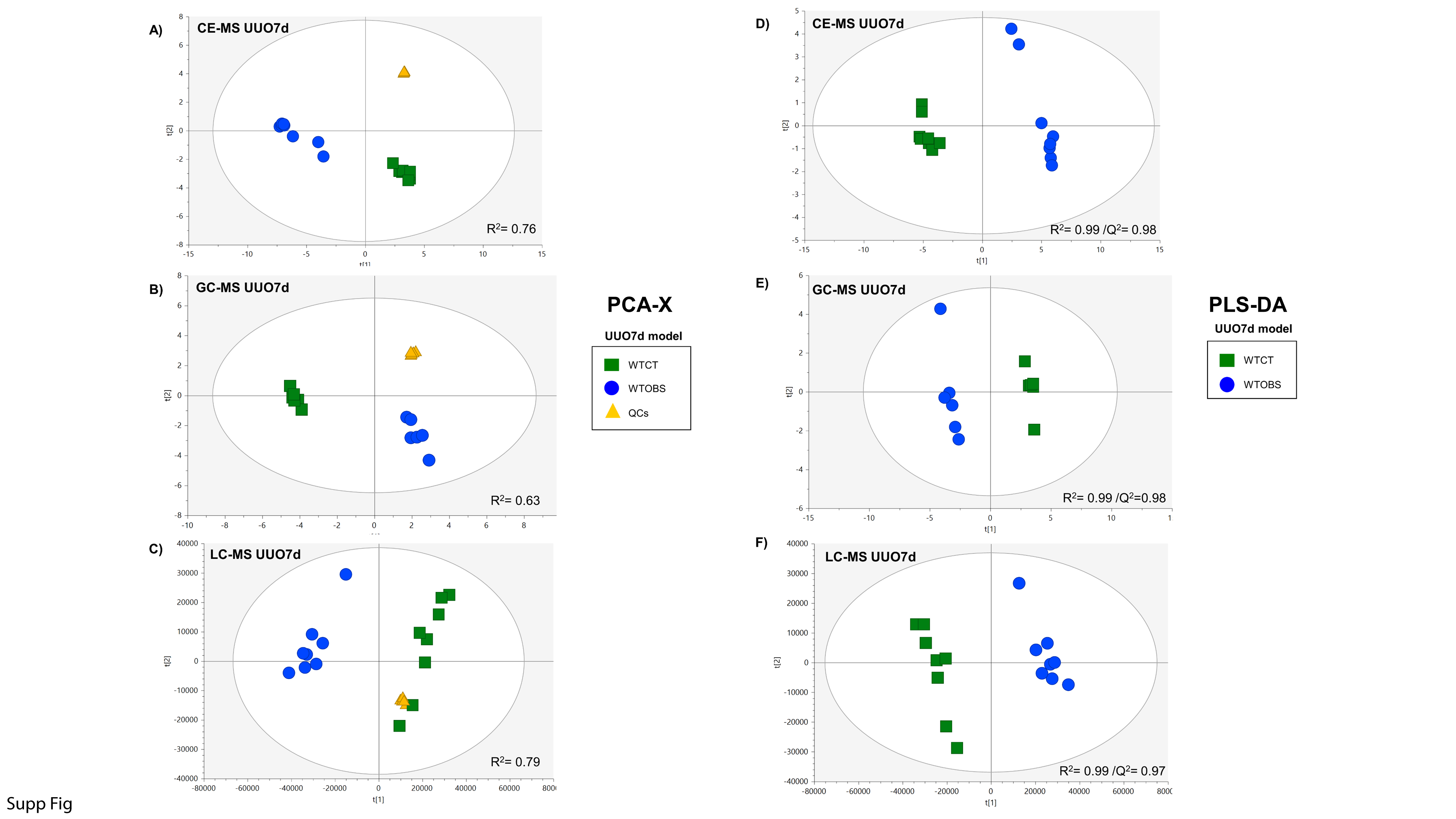

Supplement: Supplementary file 1 [file ijms-26-04933-s001.zip › UUO7d_SupplementalImage.tif]
